# Glucocorticoid receptor activation reduces food intake independent of hyperglycemia in zebrafish

**DOI:** 10.1038/s41598-022-19572-z

**Published:** 2022-09-20

**Authors:** Niepukolie Nipu, Femilarani Antomagesh, Erin Faught, Mathilakath M. Vijayan

**Affiliations:** Department of Biological Sciences, University of Calgary, Calgary, AB T2N1N4 Canada

**Keywords:** Zoology, Animal physiology

## Abstract

Chronic cortisol exposure suppresses food intake in fish, but the central mechanism(s) involved in appetite regulation are unclear. Stress and the associated increase in cortisol levels increase hepatic gluconeogenesis, leading to hyperglycemia. As hyperglycemia causes a reduction in food intake, we tested the hypothesis that cortisol-induced hyperglycemia suppresses feeding in zebrafish (*Danio rerio*). We first established that stress-independent hyperglycemia suppressed food intake, and this corresponded with a reduction in the phosphorylation of the nutrient sensor, AMP-activated protein kinase (AMPK) in the brain. Chronic cortisol exposure also led to hyperglycemia and reduced food intake, but the mechanisms were distinct. In cortisol-exposed fish, there were no changes in brain glucose uptake or AMPK phosphorylation. Also, the phosphorylation of Akt and mTOR was reduced along with an increase in *redd1*, suggesting an enhanced capacity for proteolysis. Loss of the glucocorticoid receptor did not rescue cortisol-mediated feeding suppression but did increase glucose uptake and abolished the changes seen in mTOR phosphorylation and *redd1* transcript abundance. Taken together, our results indicate that GR activation enhances brain proteolysis, and the associated amino acids levels, and not hyperglycemia, maybe a key mediator of the feeding suppression in response to chronic cortisol stimulation in zebrafish.

## Introduction

In fish, chronic stress generally leads to a decrease in food intake, and this has been attributed to the increase in circulating glucocorticoid levels post-stress^1^. The rise in glucocorticoid levels involve the coordinated stimulation of the hypothalamic-pituitary-interrenal axis (HPI) axis in fish, which is analogous to the HPA axis in mammals^2^. The hypothalamus secretes corticotropin-releasing hormone (CRH), which acts on the anterior pituitary to release proopiomelanocortin (POMC), which is cleaved by prohormone convertase 1 (PC1) to release adrenocorticotropic hormone (ACTH) into the circulation. ACTH binds to the melanocortin 2 receptor (MC2R) on the steroidogenic cells in the interrenal tissue to stimulate the biosynthesis of glucocorticoids^2,3^. In teleost, cortisol is the primary glucocorticoid released in response to stress, and is essential for the metabolic adjustments to regain homeostasis^3–5^.

A key metabolic role for cortisol during stress is elevating circulating glucose levels by increasing hepatic gluconeogenesis to fuel the increased energy demand^3–7^. This is accompanied by an enhanced muscle protein degradation, which increase the availability of amino acids as substrates for gluconeogenesis in the liver^3,6,8^. Cortisol action is mediated by two types of receptors, the low-affinity glucocorticoid receptor (GR) and the high-affinity mineralocorticoid receptor (MR), which are ligand-bound transcription factors^3,5^. Consequently, resting cortisol levels activate the MR, while the stress-induced and/or circadian elevation in cortisol levels activate the GR^3^. However, recent studies also suggest a possible interaction between the two receptors in mediating stress-related behavioural and metabolic outcomes in fish^8–10^.

Although chronic cortisol elevation has been associated with feeding suppression in fish^1^, the molecular mechanisms are not well established. Chronic cortisol exposure increases circulating glucose levels^3,6^, and, therefore, we hypothesized that stress-induced hyperglycaemia may limit food intake by increasing glucose sensing in the brain^11^. As in mammals, hyperglycemia suppresses feeding in fish, and this corresponds with a reduced phosphorylation of the AMP-activated protein kinase (AMPK), a key energy sensor^12–16^, in the hypothalamus. Together, these results suggest a role of elevated glucose levels in the cortisol-induced inhibition of feeding, but this has yet to be explicitly tested in fish^1^. Teleosts, in general, are not as adept as mammals at glucose regulation, often being considered glucose intolerant. However, it is unclear why this is the case, as insulin and its receptors are conserved in fish^17,18^. Recent studies have shown that stress levels of cortisol may limit skeletal muscle glucose uptake and peripheral insulin action in zebrafish (*Danio rerio*)^8,19^, but whether stress affects central glucose regulation is unclear in fish^20^.

In this study, we tested the hypothesis that GR activation by cortisol causes hyperglycemia and enhances glucose uptake and metabolism in zebrafish brain, leading to the feeding suppression. To address this, we first investigated whether hyperglycemia alone, in the absence of high cortisol, affected brain metabolism and feeding. To accomplish this, zebrafish were exposed to waterborne glucose concentration as described previously^21,22^. Next, we chronically elevated cortisol content in zebrafish by waterborne exposure to this steroid as described previously^9^. We assessed whether this treatment led to hyperglycemia and mimicked the associated changes in brain metabolism and feeding. Finally, to discern whether the effects of cortisol were mediated by GR activation, we utilized a ubiquitous GR knockout zebrafish^9^. Also, to further confirm GR activation, as well as to test whether MR may play a role, we also carried out feeding trials with zebrafish lacking MR^9^. Following the treatments, the brain capacity for glucose uptake, as well as the transcript abundance (*slc2a*) of glucose transporters (GLUTs) and the phosphorylation of AMPK, a key cellular energy sensor^15^, were ascertained. Further assessment of the brain metabolic changes examined the insulin/growth factors signalling pathway, including insulin expression, their receptors transcript abundance, and the downstream phosphorylation of Akt and mTOR expression. We also measured the transcript abundance of regulated in development and DNA damage responses 1 *(redd1)*, a key GR-responsive gene and a marker of protein breakdown, as well as an mTOR regulator^8,23–25^. Additionally, the transcript abundance of several key regulatory peptides related to feeding, including *npy, crh, pomca, mc3r, mc4r,* and *lepa*, were also measured. Overall, our results indicate that GR mediates the feeding suppression seen with cortisol, and this is due to alterations in the brain energy metabolism in zebrafish.

## Materials and methods

### Animal husbandry

All adult zebrafish (Tupfel long fin strain) were maintained in 10 L tanks on a recirculating system held at a 14:10 light: dark cycle (Pentair Aquatic habitats, Apopka, Fl, USA). Water temperature was maintained at 28.5 °C with pH and conductivity at 7.6 and 750-µS, respectively. Animals were fed with Gemma micro 300 (Skretting, USA) in the morning and live Artemia (San Francisco Bay Brand, USA) in the afternoon. The animal care protocol (AC17-0079) was approved by the University of Calgary Animal Care Committee, and followed the guidelines set by the Canadian Council on Animal Care.The fish lacking GR (GRKO) and MR (MRKO) zebrafish lines were generated as previously described using CRISPR/Cas9 mutagenesis. Briefly, GRKO fish have a − 7 bp deletion (nr3c1^ca401/ca401^) and the MRKO fish have a + 8 bp insertion (nr3c2^ca402/ca402^). Wildtype (WT) fish were a result of an F1 heterozygous incross and all knockout fish used were maternal zygotic mutants (F3 generation). We have reported the study in accordance with the ARRIVE guidelines.

### Glucose treatment

Age-matched adult zebrafish (2:1 male to female ratio) were transferred from a recirculatory system to a 2 L freshwater static system (8 fish/tank) with aeration. The glucose treatment followed the protocol described previously^21,22^, with slight modification. We carried out a preliminary study to assess the waterborne glucose concentration (111 mM and 278 mM) required to maintain hyperglycemia after an overnight exposure. Briefly, at 17:00 h fish were moved to static tanks and treated with either no glucose (control) or 278 mM glucose in the water. The following morning (10:00 h) fish were either sampled or used for the feeding trials as described below.

### Cortisol treatment

Age-matched adult WT zebrafish (1:1 male to female ratio) were transferred from a recirculatory system to a 2 L freshwater static system (8 fish/tank), with aeration, and treated with cortisol at a concentration of 10 µg/mL as described previously with minor modification^24^. Cortisol was dissolved in ethanol with a final concentration of 0.05%, and this was also maintained in the control tanks. Briefly, at 17:00 h fish were moved to static tanks with aeration and treated with either cortisol or the vehicle. The following morning (10:00 h) fish were either sampled or used for the feeding trials as described below. For the 2-[*N*-7-nitrobenz-2-oxa-1,3-diazol-4-yl) amino-2-deoxy-d-glucose (2-NBDG) study, adult WT zebrafish (1:1 male to female ratio) were transferred from a recirculatory system to a 2 L freshwater static system (6–8 fish/tank), with aeration, and exposed to either vehicle (0.05% ethanol) or cortisol (5 µg/mL) for 3 d. The fish were fed twice with Gemma micro 300 (Skretting, USA) and a 100% water exchange was carried out every 24 h.

### 2-NBDG uptake

Brain 2-NBDG uptake study was carried out with adult fish treated with cortisol (see above), and also with the WT and GRKO zebrafish^8^. As the GRKO fish (3:1 male to female ratio) are inherently hypercortisolaemic due to a loss of negative feedback^9^, we did not treat that genotype with cortisol. For the cortisol study, after 3 d of exposure, the control and cortisol-treated adult zebrafish were intraperitoneally injected with 0.05 µmol 2-NBDG, as previously described^8^, along with either saline or insulin (0.0075 U/ g). The exact same procedure was also carried out for the wildtype and GRKO comparison, but without the insulin injection. The injected fish were euthanized after a 1 h recovery period and the whole brain was immediately removed and homogenized in 50 mM Tris-Buffer (pH 7.5) with a protease inhibitor cocktail (Roche Diagnostics, USA) using a sonicator (Fisher Scientific, 3 × 3 s pulse). Samples were centrifuged at 13,000×*g* for 1 min, and the supernatant was added to a black 96 well plate and the relative fluorescence unit measured using the Paradigm plate reader (Molecular devices, USA). The fluorescence was measured at 465/540 nm excitation/emission wavelength as described previously^8^.

### Feeding performance

Feeding performance was carried out with the glucose and cortisol treatments described above, and also with the WT and GRKO and WT and MRKO (1:1 male to female ratio) fish. As MRKO fish have normal cortisol levels^9^, additional treatment with exogenous cortisol was necessary to assess whether MR or GR mediated the effects of this hormone. Briefly, at 17:00 h fish (WT and MRKO) were moved to static tanks with aeration and treated with either cortisol 5 µg/mL or the vehicle (0.05% ethanol). The following morning (10:00 h) feeding performance was assessed as described previously^8^. Briefly, fish were placed individually to a 2 L transparent tank with 1 L of system water and fed ten pellets at a time. The pellets were counted at 5-min intervals, new pellets provided if all had been consumed, for a total of 20 min. Fish were euthanized with MS-222 (buffered 1:2 with NaHCO_3_) either before or after the feeding trial, and the brain and liver stored at − 80 °C for further analysis.

### Cortisol and glucose determination

Whole-body cortisol was determined after diethyl ether extraction as previously described^26^. Cortisol was measured using an ELISA that was validated for zebrafish^26^. Blood glucose was quantified using FreeStyle glucose strips and meter (Abbott, Mississauga, ON, Canada), which was validated for zebrafish^27^.

### Immunodetection

SDS-PAGE, Western blotting, and dot blot were performed as previously described^8,9^. Briefly samples were homogenized in 50 mM Tris-Buffer (pH 7.5) with a protease inhibitor cocktail (Roche Diagnostics, USA) using a sonicator (Fisher Scientific, 3 × 3 s pulse; ~ 30% power), after which the samples were centrifuged at 13,000×*g* for 2 min. Protein concentration of the supernatant was determined using the bicinchoninic acid (BCA) method with bovine serum albumin as standards. Samples were diluted in 5 × Laemmli’s buffer (156.25 mM Tris, 50% glycerol, 5% SDS, 0.0625% bromophenol blue and 25% 2-mercaptoethanol) and stored at − 20 °C. For the dot blot, 2 μL of sample (2 mg/mL protein) was added to a nitrocellulose membrane. The membrane was then dried for 2 h at 37 °C. Western blots were performed by separating equal amounts of protein (40 μg) on a polyacrylamide gel (8%), and then transferred to a nitrocellulose membrane with a SemiDry transfer unit (BioRad). After transfer, the membranes were blocked for 1 h in 5% skim milk at room temperature, and this was followed by an overnight incubation at 4 °C with the appropriate antibody (see below), that have been previously used with zebrafish tissues^8,9,28–30^. The primary antibodies used are as follows: anti-phospho-mTOR (mammalian target of rapamycin) [Cell Signaling Technology, # 2971, 1:1000]; anti-phospho-Akt (Ser473) (Protein Kinase B) [Cell Signaling Technology, # 9271, 1:1000]; anti-Akt (Protein Kinase B) [Cell Signaling Technology, # 9272, 1:1000]; anti-Phospho-AMPKα (Thr172)(AMP-activated protein kinase) [Cell Signaling Technology, # 2535, 1:1000]; anti-AMPKα (AMP-activated protein kinase) [Cell Signaling Technology, # 5831, 1:1000]; anti-insulin [Agilent Technologies, # A056401-2, 1:500]; CY3 conjugated anti-β-actin (Sigma; C5838 1:1000) Immunodetection was carried out as previously described^8,9^. This insulin antibody works well with zebrafish as described previously^8,31^. Following overnight incubation with the primary antibody, membranes were washed with TTBS (5 min, 3×) and incubated for 1 h with secondary antibody (1:3000 Goat anti-rabbit IgG; Bio-Rad, 170-6515 or 1:3000 Goat anti-Guinea Pig IgG (H/L):HRP for anti-insulin; Bio-Rad, AHP863P). Using Clarity Western ECL substrate (BioRad, 170-5061), protein bands were detected. Relative band intensity was quantified using ImageJ software (National Institutes of Health, Bethesda, MD) as described previously^8,9^.

### Transcript abundance

The transcript abundance of specific genes was measured by quantitative real-time PCR (qPCR). Total RNA was extracted from the brain and liver using Ribozol (VWR) according to the manufacturer’s instructions. Using a Spectradrop Micro-Volume Microplate (VersaMax, Molecular Devices, CA, USA), RNA was quantified, and 1 μg of total RNA was treated with DNase I (Thermo Scientific, Waltham, MA, USA) before cDNA synthesis using the high-capacity cDNA reverse transcription kit (Applied Biosystems, Foster City, CA, USA). Quantitative PCR was carried out as previously described^26^ using a QuantStudio 3 Real-Time PCR system (Applied Biosystems), with SsoAdvanced™ Universal SYBR® Green Supermix (Biorad, Canada). All samples were run in duplicates using gene-specific primers (Supplemental information Table S1). Samples were run with the following cycling conditions: 94 °C for 2 min, 40 cycles of 95 °C for 30 s, and 30 s at 60 °C, and a final extension step at 72 °C for 10 min. The relative expression of the transcripts were quantified using the 2^−∆∆Ct^ method^32^ and amplicon specificity was confirmed by melt curve analysis. β-actin was used as the housekeeping gene for normalization and the cycle thresholds (Cts) for this gene did not change between treatments.

### Statistics

All analysis was carried out using Sigma Plot 14 (Systat Software Inc., San Jose, USA) and the graphs were plotted using Graphpad Prism 9.2.0 (Graphpad software, USA). The data are shown as means ± SEM. The two sample comparisons were carried out using an unpaired *t* test (*P* < 0.05). The transcript abundance of *slc2a* were analysed using one-way ANOVA (*P* < 0.05; Holm-Sidak post hoc), insulin glucose uptake and MRKO feeding data was analysed using a two-way ANOVA (*P* < 0.05; Holm-Sidak post hoc). Data was transformed to meet the assumptions of normality and equal variance or analysed using the Mann–Whitney *U* test. Non-transformed values are shown in the figures.

## Results

### Glucose exposure inhibits food intake

**Figure 1:**
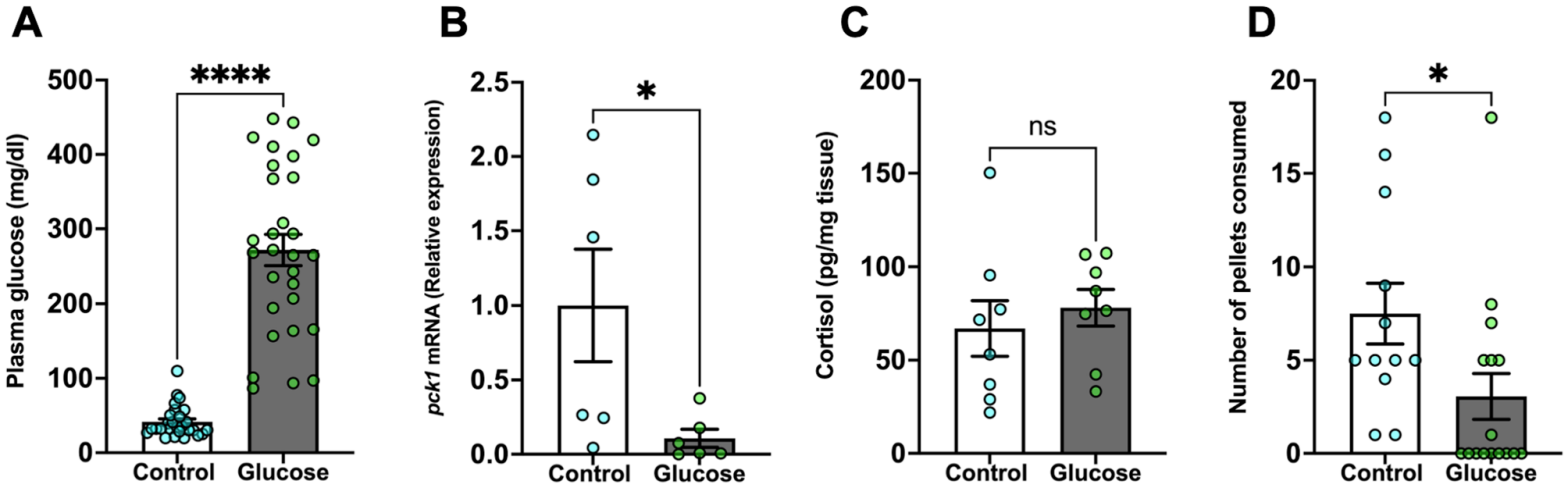
Glucose exposure inhibits food intake. (**A**) Plasma glucose was significantly higher in the glucose-treated fish compared to the control fish. (**B**) *pck1* transcript abundance in the liver significantly decreased after glucose treatment compared to the control fish. (**C**) Whole-body cortisol levels remained unchanged in the glucose-treated fish compared to the control fish. (**D**) Glucose-treated fish consumed significantly fewer pellets than the control. Values are means ± SEM. (Plasma glucose n = 27–29; Transcript abundance n = 6; Whole-body cortisol n = 8; Feeding performance n = 12–16). ns—not significant; *(*P* < 0.05); ****(*P* < 0.0001)).

Glucose-treated fish had significantly higher blood glucose levels compared to the control group (Fig. 1A; *P* < 0.0001). We then measured the liver transcript abundance of phosphoenolpyruvate carboxykinase (*pck1*), which is a key gluconeogenic gene essential for maintaining glucose homeostasis^33^. The transcript abundance of *pck1* in the liver was significantly lower in the glucose-treated fish compared to the control fish (Fig. 1B; *P* = 0.041). Glucose treatment did not affect whole-body cortisol levels when compared to the control fish (Fig. 1C; *P* = 0.544). The food intake in the glucose-treated fish was significantly lower, and they ate less than half of the food consumed by the control fish (Fig. 1D; *P* = 0.012).

### Glucose reduces AMPK phosphorylation in the brain

**Figure 2:**
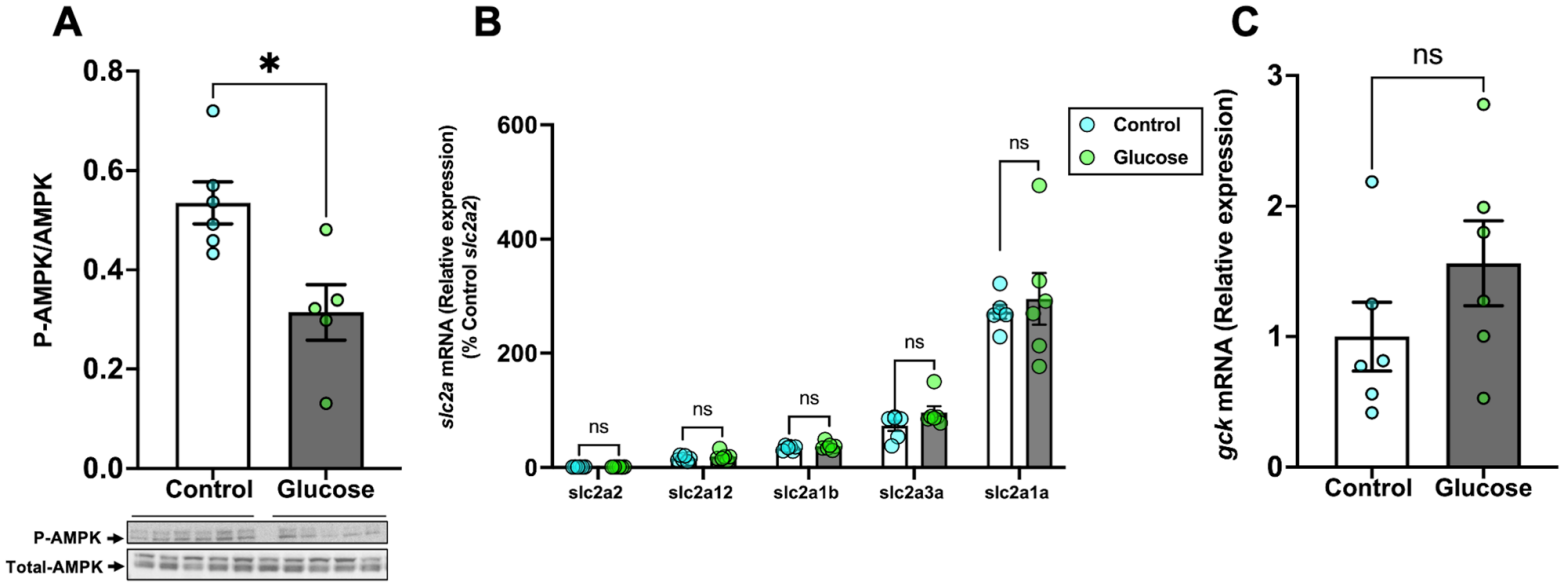
Glucose reduces AMPK phosphorylation in the brain. **(A**) Glucose exposure decreased the ratio of phosphorylated to total AMPK (M.W.:62 kDa) in the brain compared to the control. (**B**) The transcript levels of the glucose transporters (*slc2a*’s) in the brain did not change after glucose exposure. As we were comparing relative abundance of all *slc2a*’s in the brain, we chose to normalize all *slc2a*’s to the *slc2a*2, which showed the lowest transcript abundance. (**C**) mRNA levels of glucokinase (*gck*) remained unchanged after glucose exposure when compared to the control. Values are means ± SEM. (AMPK expression n = 5–6; Transcript abundance n = 6). ns—not significant; *(*P* < 0.05).

To test if the decrease in food intake corresponded with the phosphorylation of AMPK, a crucial energy sensor regulating appetite^15^, we measured the ratio of phosphorylated to total AMPK expression in the brain. The AMPK expression was significantly lower in the glucose-treated fish compared to the control fish (Fig. 2A; *P* = 0.011 and also see Figs. S6A and S6D). We assessed if the glucose treatment altered the transcript abundance of the GLUTs, including *slc2a1a, slc2a1b, slc2a2, slc2a3a* and *slc2a12.* The transcript levels of *slc2a1a* (*P* = 0.64)*, slc2a1b* (*P* = 0.344)*, slc2a2* (*P* = 0.906)*, slc2a3a* (*P* = 0.129) and *slc2a12* (*P* = 0.383) (Fig. 2B) in the brain were not significantly different between the two groups. We also measured the transcript abundance of glucokinase (*gck*) which is known to be involved in brain glucose-sensing^11^.The transcript levels of *gck* remained unaltered in the glucose treated fish when compared to the control fish (Fig. 2C; *P* = 0.21). The steady state transcript abundance of brain appetite regulating peptides, including *npy* (Supplemental: Fig. S1A; *P* = 0.136), *crh* (Supplemental: Fig. S1B; *P* = 0.106), *pomca* (Supplemental: Fig. S1C; *P* = 0.21), *mc3r* (Supplemental: Fig. S1D; *P* = 0.836, *mc4r* (Supplemental: Fig. S1E; *P* = 0.246), and *lepa* (Supplemental: Fig. S1F; *P* = 0.653) were not significantly different in the glucose-treated fish compared to the control fish.

### Glucose did not affect Akt and mTOR phosphorylation

**Figure 3:**
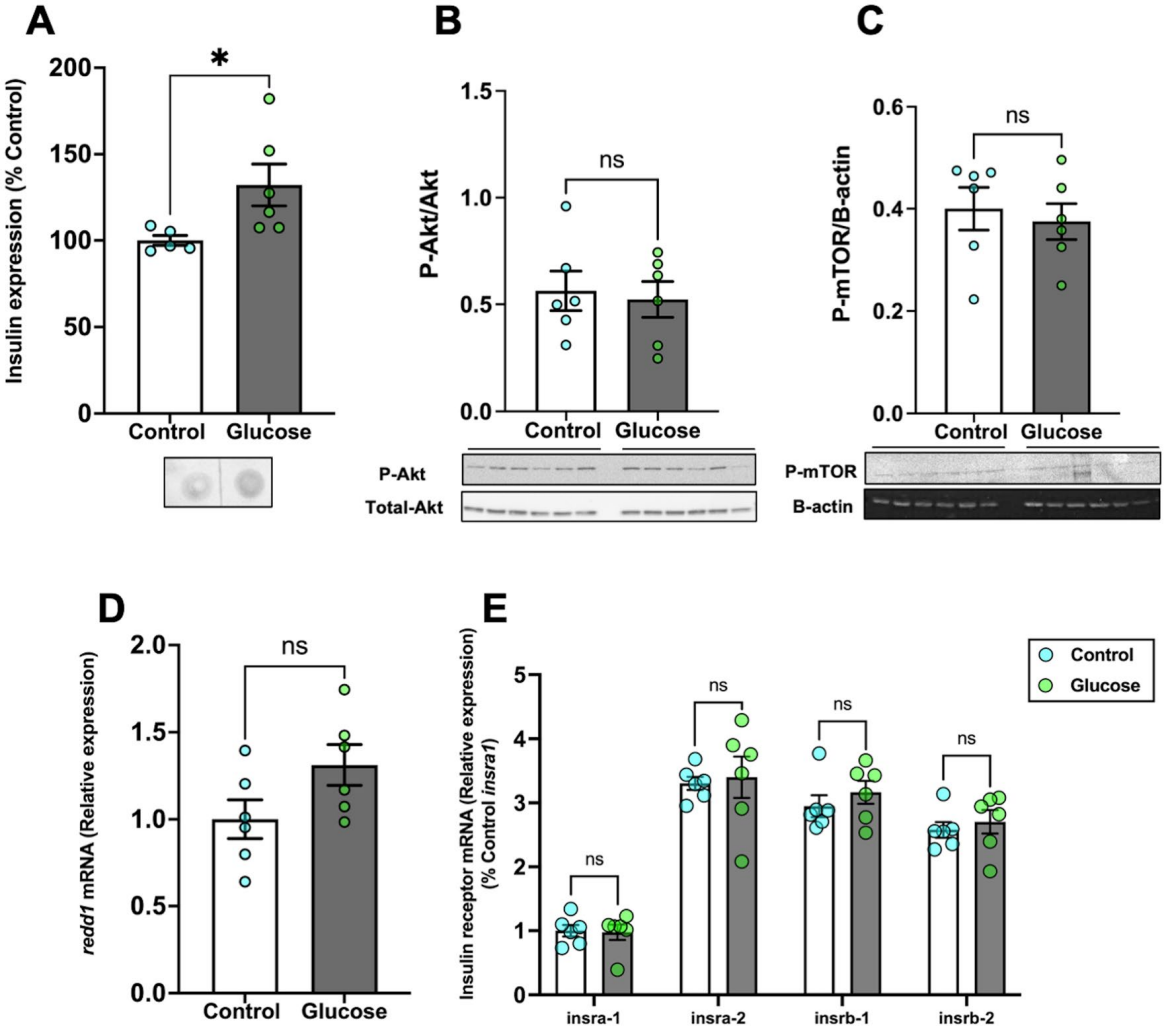
Glucose did not affect Akt and mTOR phosphorylation. (**A**) Whole-body insulin expression significantly increased after glucose exposure compared to the control. (**B**) Glucose exposure did not alter the ratio of phosphorylated to total Akt (M.W.:60 kDa) in the brain when compared to the control. (**C**) The phosphorylation of mTOR (M.W.:289 kDa) also remained unchanged in the glucose-treated fish compared to the control. (**D**) Brain *redd1* mRNA levels remained unchanged in the glucose-treated fish compared to the control. (**E**) The transcript levels of the insulin receptor genes in the brain did not change after glucose treatment. Values are means ± SEM. (Whole-body insulin n = 5–6; Protein expression n = 6; Transcript abundance n = 6). ns—not significant; *(*P* < 0.05).

We tested if glucose affected insulin expression and the Akt-mTOR signalling pathway. Glucose exposure significantly increased the expression of whole-body insulin compared to the control (Fig. 3A; *P* = 0.043). The ratio of phosphorylated to total Akt (Fig. 3B; *P* = 0.753) did not significantly change in the brain of the glucose-treated fish compared to the control fish. We assessed if this was also reflected downstream by changes in the phosphorylation of mTOR. Glucose treatment did not significantly alter the phosphorylation of mTOR in the brain of the glucose-treated fish compared to the control fish (Fig. 3C; *P* = 0.656). There was no significant difference in the transcript abundance of *redd1* in the brain of glucose treated fish compared to the control fish (Fig. 3D; *P* = 0.083). Also, the transcript abundance of the insulin receptors, including *insra-1* (*P* = 0.873), *insra-2* (*P* = 0.785), *insrb-1* (*P* = 0.396), *insrb-2* (*P* = 0.583), in the brain remained unchanged in the glucose-treated fish compared to the control fish (Fig. 3E).

### Cortisol suppresses feeding

**Figure 4:**
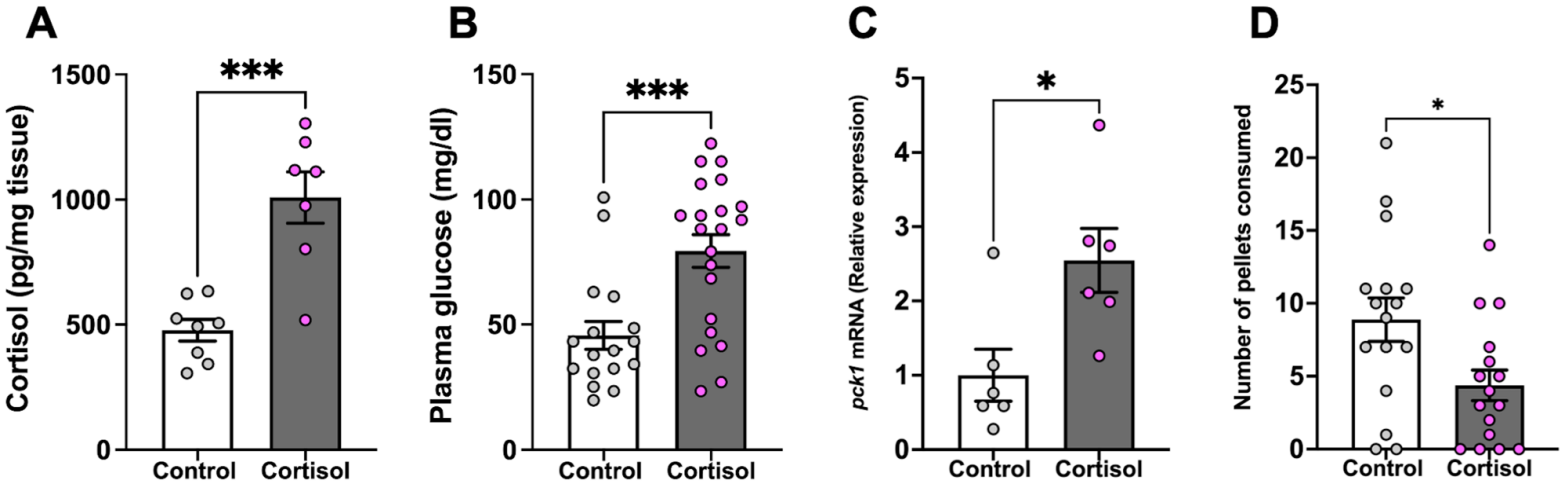
Cortisol suppresses feeding. (**A**) Whole-body cortisol levels were higher in the cortisol-treated fish compared to the control. (**B**) Cortisol treatment significantly increased plasma glucose levels. (**C**) The *pck1* transcript abundance in the liver significantly increased after cortisol treatment compared to the control. (**D**) Cortisol-treated fish consumed significantly fewer pellets than the control. Values are means ± SEM (Whole-body cortisol n = 7–8; Plasma glucose n = 17–21; Transcript abundance n = 6; Feeding performance n = 16). ns—not significant; *(*P* < 0.05); ***(*P* < 0.001).

Cortisol-treated fish had significantly higher whole-body cortisol (Fig. 4A; *P* < 0.001) and glucose levels (Fig. 4B ; *P* < 0.001) compared to the control fish. To assess if gluconeogenesis played a role in the elevated glucose level, we measured the transcript abundance of the key gluconeogenic gene *pck1* in the liver of zebrafish. The transcript abundance of *pck1* was significantly higher with a twofold increase in the cortisol-treated fish compared to the control fish (Fig. 4C; *P* < 0.02). Exposure to cortisol significantly decreased the number of pellets consumed compared to the control fish (Fig. 4D; *P* = 0.019). To assess if the reduction in food consumption reflect altered stead-state transcript abundance of feeding-related brain peptides in the cortisol group, we measured the transcript abundance of genes that encodes for appetite regulation in the brain of zebrafish. Cortisol exposure did not significantly alter the transcript abundance of *npy* (Supplemental: Fig. S2A; *P* = 0.67), *crh* (Supplemental: Fig. S2B; *P* = 0.67), *pomca* (Supplemental: Fig. S2C; *P* = 0.223), *mc3r* (Supplemental: Fig. S2D; *P* = 0.740), *mc4r* (Supplemental: Fig. S2E; *P* = 0.177), and *leptin a (lep a)* (Supplemental: Fig. S2F; *P* = 0.691) compared to the control group.

### Cortisol does not affect brain glucose uptake

**Figure 5:**
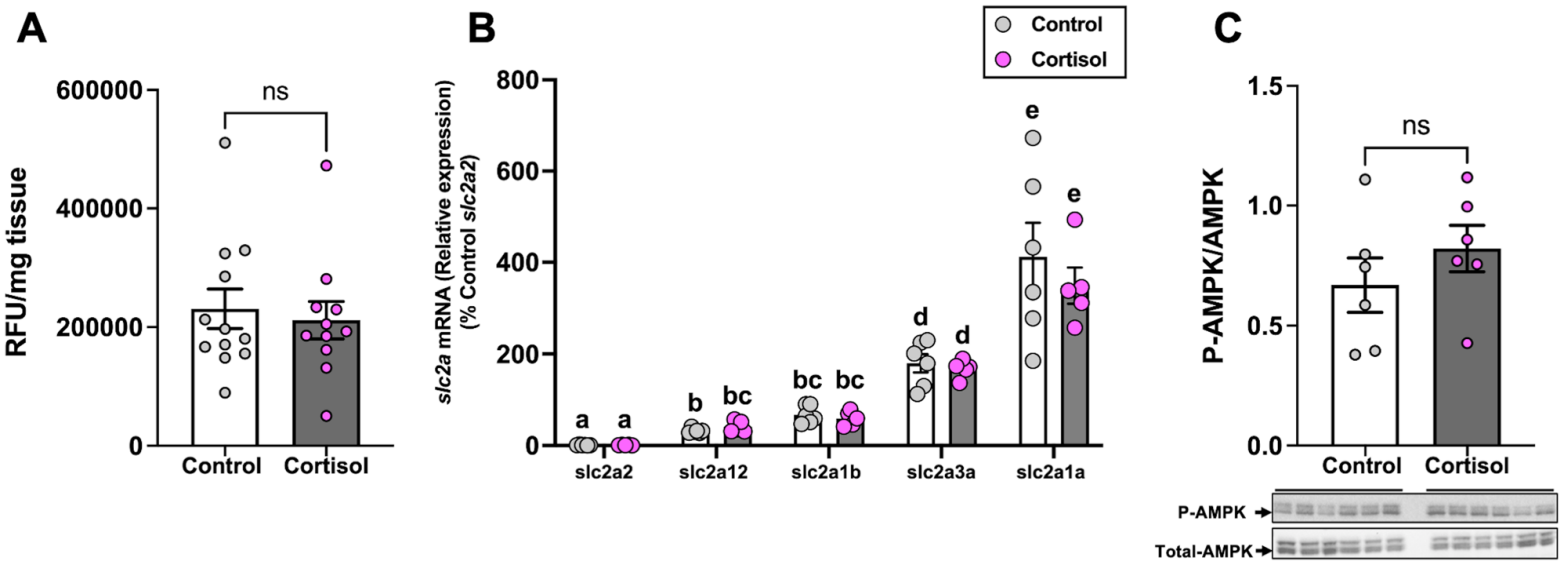
Cortisol does not affect brain glucose uptake. (**A**) Uptake of the fluorescent glucose analogue 2-NBDG in the brain of the cortisol-treated fish remained unchanged compared to the control. (**B**) The transcript levels of the glucose transporters (*slc2a*’s) in the brain did not change after cortisol exposure. (**C**) Cortisol treatment did not change the ratio of phosphorylated to total AMPK (M.W.:62 kDa) in the brain. Values are means ± SEM (Relative fluorescence units; RFU n = 11–12; Transcript abundance n = 5–6; AMPK expression n = 6). ns—not significant; Bars with different letters are significantly different (one-way ANOVA; P ≤ 0.05).

We tested if the brain capacity for glucose uptake is altered in response to cortisol and insulin treatment, and if that reflected a change in the transcript abundance of glucose transporters. There was no significant effect of cortisol exposure on brain 2-NBDG uptake compared to the control fish (Fig. 5A; *P* = 0.623). Insulin injection also did not significantly affect brain glucose uptake in both the control and cortisol treated group compared to the saline injected fish (Supplemental: Fig. S3; *P* > 0.05). The transcript abundance of *slc2a1a, slc2a1b, slc2a2, slc2a3a* and *slc2a12* were measured in the brain of zebrafish. Among the GLUTs measured, *slc2a2* had the lowest expression followed by *slc2a12, slc2a1b*, *slc2a3a* and *slc2a1a* in the brain of zebrafish (Fig. 5B; *P* = 0.05). However, no significant changes were observed in the transcript abundance of *slc2a* between the cortisol-treated fish and the control fish (Fig. 5B). Finally, to test if cortisol treatment altered cellular energy status, we measured the expression of phospho-AMPK. The ratio of phosphorylated to total AMPK expression in the brain was not significantly affected by the cortisol-treatment compared to the control fish (Fig. 5C; *P* = 0.331 and also see Figs. S6B and S6E).

### Cortisol affects brain Akt and mTOR phosphorylation

**Figure 6:**
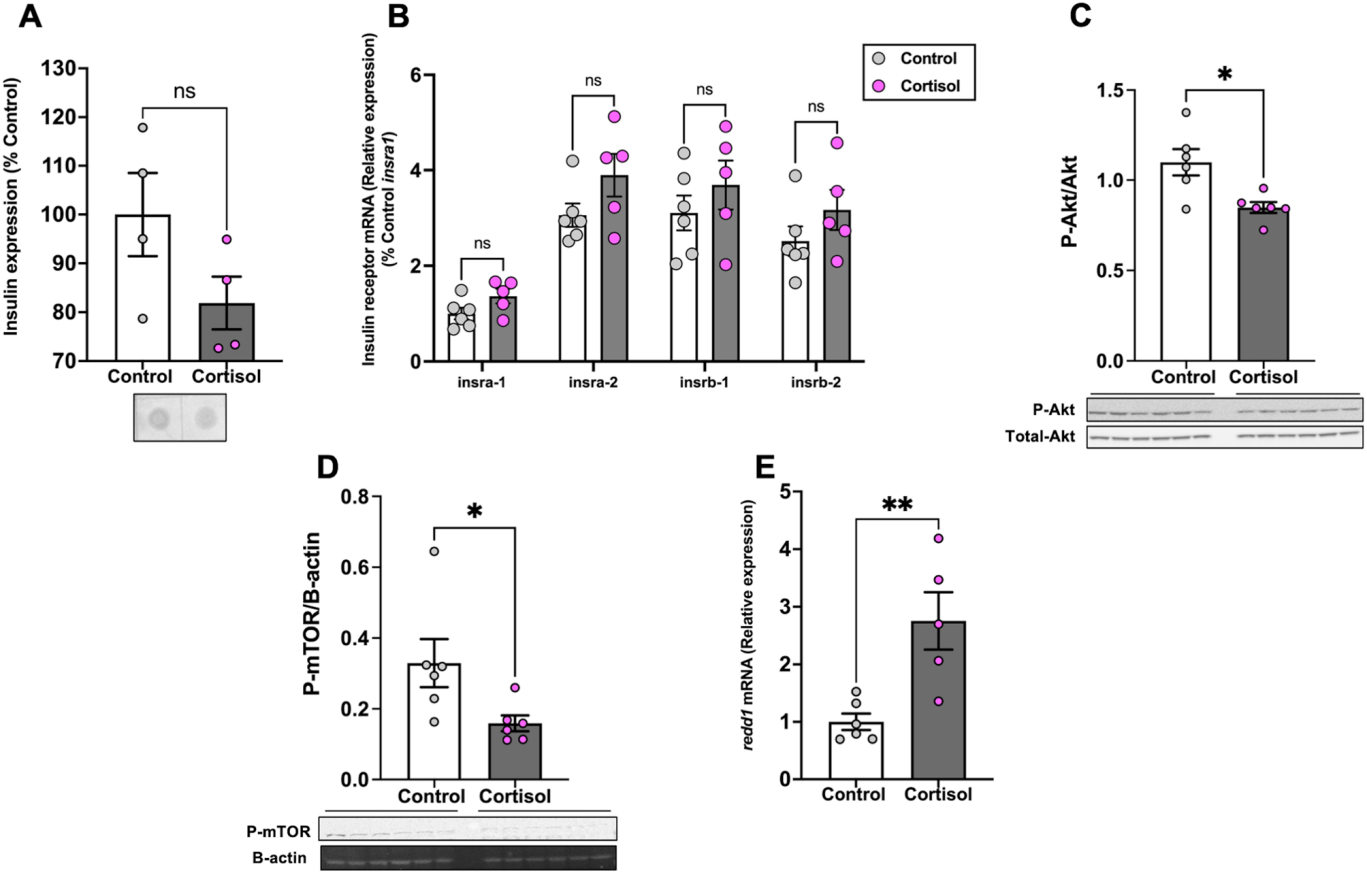
Cortisol affects brain Akt and mTOR phosphorylation. (**A**) Cortisol treatment did not change whole-body insulin expression levels. (**B**) We observed no changes in the transcript levels of the insulin receptor genes in the brain after cortisol treatment. (**C**) The ratio of phosphorylated to total Akt (M.W.:60 kDa) in the brain was significantly lower in the cortisol-treated fish when compared to the control. (**D**) The phosphorylation of mTOR (M.W.:289 kDa) was also significantly lower after cortisol treatment. E. *redd1*mRNA levels significantly increased in the brain of the cortisol treated group compared to the control. Values are means ± SEM (Whole-body insulin n = 4; Protein expression n = 6; Transcript abundance n = 5–6). ns—not significant; *(*P* < 0.05); **(*P* < 0.01).

To test if cortisol affects Akt-mTOR pathway, we measured the whole-body expression of insulin, as well as their receptors and key signalling markers, including Akt and mTOR phosphorylation in the brain of zebrafish. Cortisol did not significantly affect whole-body insulin expression compared to the control group (Fig. 6A; *P* = 0.122). Also, cortisol did not significantly affect the transcript abundance of the insulin receptors, including *insra-1* (*P* = 0.088), *insra-2* (*P* = 0.118), *insrb-1* (*P* = 0.367) and *insrb-2* (*P* = 0.232) in the brain of zebrafish compared to the control group (Fig. 6B). However, cortisol exposure significantly reduced the ratio of phosphorylated to total Akt in the brain of zebrafish compared to the control group (Fig. 6C; *P* = 0.010). Additionally, the cortisol-treated fish had significantly lower expression of the phospho-mTOR compared to the control group (Fig. 6D; *P* = 0.039). To test if elevated cortisol levels affect brain protein breakdown, we measured the transcript abundance of *redd1*, an inhibitor of mTOR signalling and a marker of proteolysis^34^. Cortisol exposure significantly increased the transcript abundance of *redd1* in the brain of zebrafish compared to the control fish (Fig. 6E; *P* = 0.003).

### Lack of GR suppresses food intake

**Figure 7:**
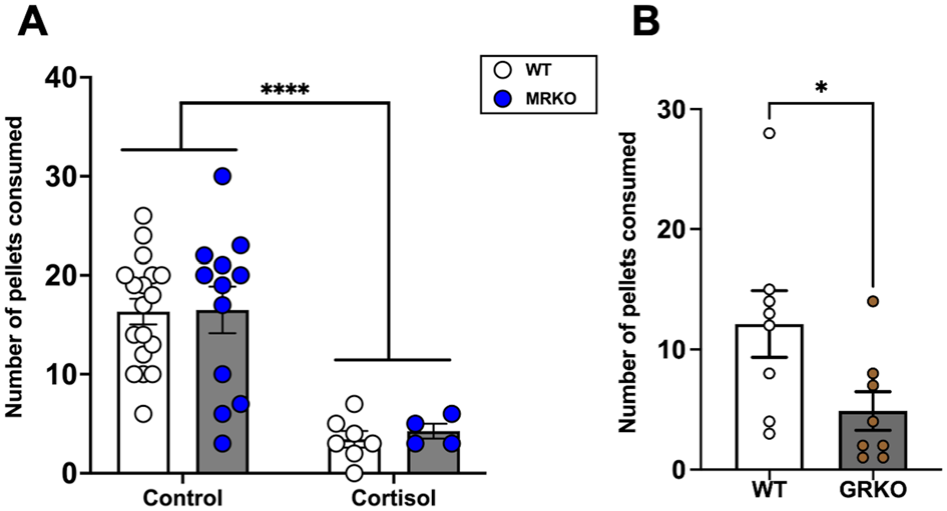
Loss of GR suppresses food intake. (**A**) MRKO fish lacks MR, while GR activation by cortisol treatment reduces the food consumed (control groups of both genotype had 12–18 fish and the cortisol group had 4–7 fish). *Significantly different from the control (2-way ANOVA; *P* < 0.001). (**B**) In fish lacking GR (GRKO) also the food intake was reduced (n = 8). Values are means ± SEM. ns—not significant; *(*P* < 0.05); ****(*P* < 0.0001).

To test if cortisol-GR activation influenced food intake, WT and MRKO fish were subjected to feeding trials before and after exposure to exogenous cortisol. The food intake did not significantly change between the WT and MRKO fish before cortisol treatment (Fig. 7A). However, after cortisol exposure, both WT and MRKO mutants had a significant reduction in food intake compared to the control (untreated group) (Fig. 7A; *P* < 0.0001), suggesting that cortisol-GR activation suppresses appetite. To further confirm if the lack of GR also suppressed feeding, we carried out feeding trials with the GRKO fish^8^. Fish lacking GR were hypercortisolemic (Supplemental: Fig. S4A; *P* = 0.04), while the blood glucose levels (Supplemental: Fig. S4B; P = 0.162) and the liver *pck1* transcript abundance remained unchanged (Supplemental: Fig. S4C; *P* = 0.093) between the two genotypes. There was a significant reduction in food consumption in the fish lacking GR compared to the wildtype (Fig. 7B; *P* = 0.040). There was no significant change in the mRNA transcript levels of *npy* (Supplemental: Fig. S5A; *P* = 0.062), *crh* (Supplemental: Fig. S5B; *P* = 0.074), *pomca* (Supplemental: Fig. S5C; *P* = 0.995), *mc3r* (Supplemental: Fig. S5D; *P* = 0.094), *mc4r* (Supplemental: Fig. S5E; *P* = 0.346). However, the GRKO fish had decreased *lepa* expression compared to the WT (Supplemental: Fig. S5F; *P* = 0.044).

### Loss of GR increases brain glucose uptake

**Figure 8:**
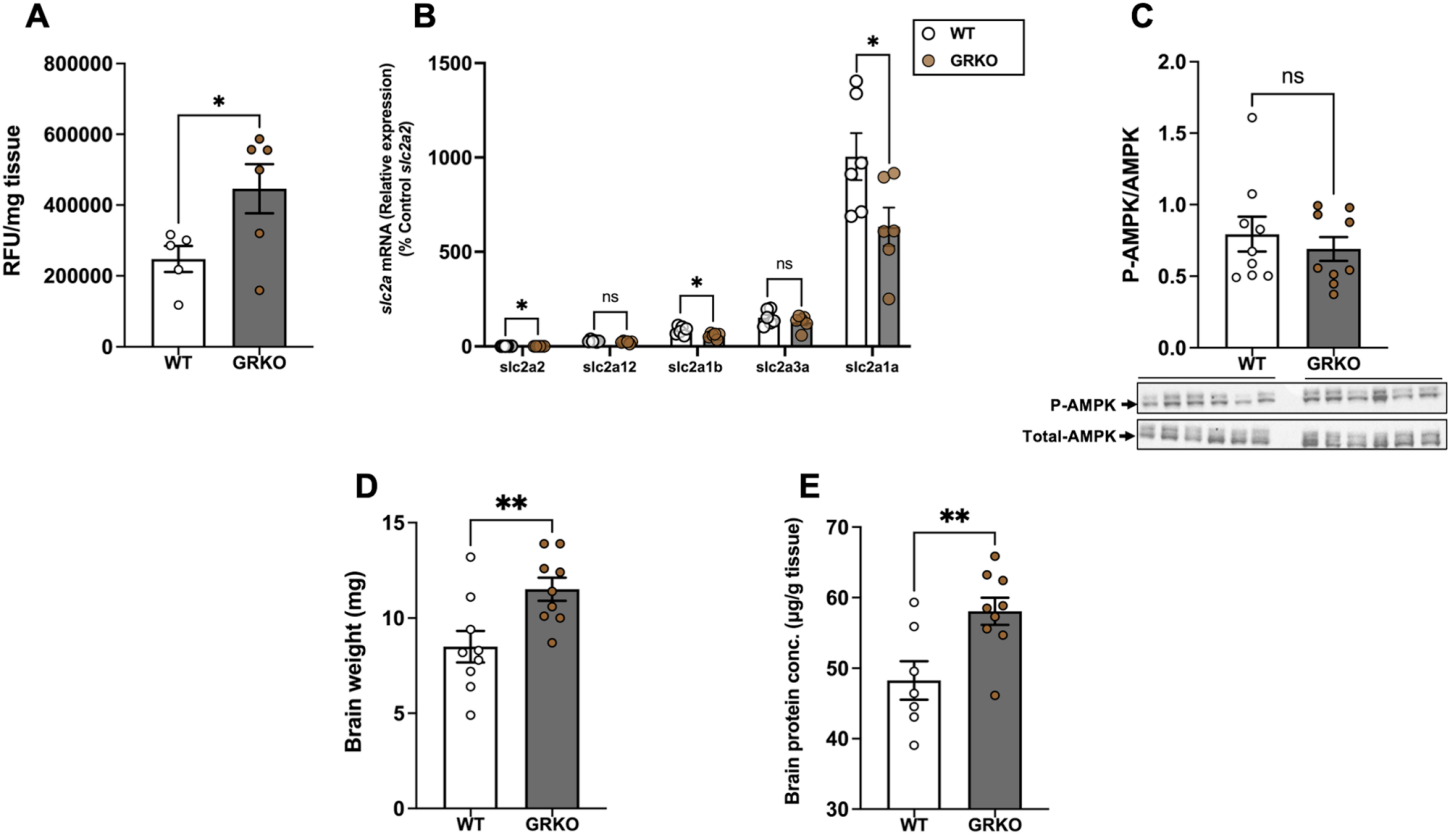
Loss of GR increases brain glucose uptake. (**A**) GRKO fish had significantly higher uptake of the fluorescent glucose analogue 2-NBDG in the brain than the WT. (**B**) The transcript levels of *slc2a1a*, *slc2a1b* and *slc2a2* in the brain was lower in the GRKO fish compared to the WT, but the expression of *slc2a3a* and *slc2a12* remained unchanged. (**C**) Loss of GR did not change the ratio of phosphorylated to total AMPK (M.W. 62 kDa) in the brain. (**D**) GRKO fish had significantly higher brain mass compared to the WT. E. Brain protein concentration was significantly higher in the GRKO fish compared to WT. Values are means ± SEM (Relative fluorescence units; RFU n = 5–6; Transcript abundance n = 6; AMPK expression n = 9; Brain wet weight n = 9; Brain protein concentration n = 7–9). ns—not significant; *(*P* < 0.05); **(*P* < 0.01).

The glucose uptake capacity, as measured by 2-NBDG uptake, was significantly higher in the GRKO fish compared to the wildtype (Fig. 8A; *P* = 0.042). The transcript abundance of *slc2a2* (*P* = 0.017), *slc2a1b* (*P* = 0.023) and *slc2a1a* (*P* = 0.044) were significantly lower in the GRKO fish, but *slc2a12* (*P* = 0.093) and *slc2a3a* (*P* = 0.314) remained unchanged compared with the WT (Fig. 8B). There was no significant difference in the ratio of phosphorylated to total AMPK expression in the brain of GRKO fish compared to the WT brain (Fig. 8C; *P* = 0.515 and also see Figs. S6C and S6F). The GRKO fish had significantly higher brain mass (Fig. 8D; *P* = 0.01) and total protein concentration (Fig. 8E; *P* = 0.009) compared to the WT.

### Loss of GR affects brain Akt phosphorylation

**Figure 9:**
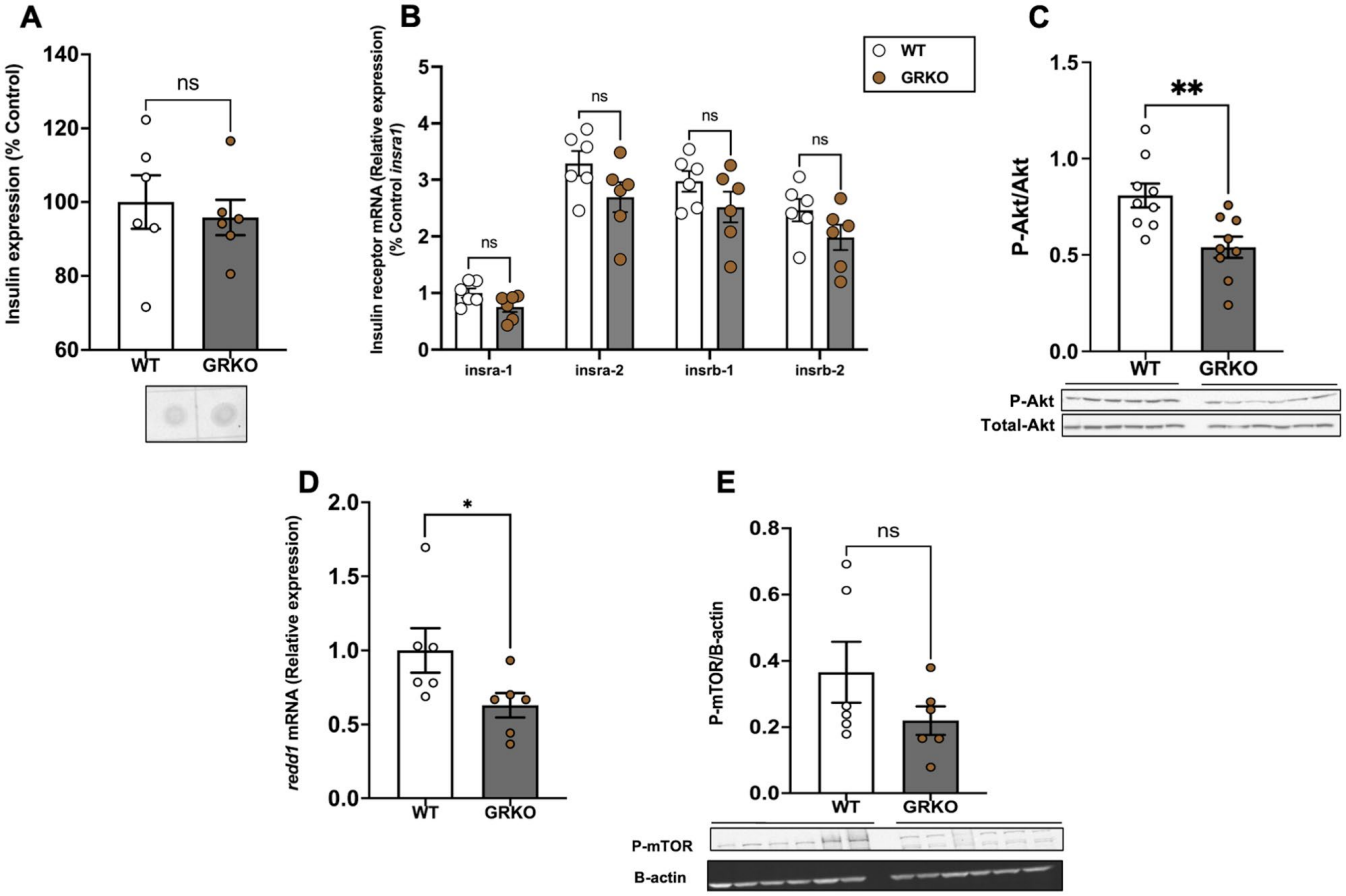
Loss of GR affects brain Akt phosphorylation. (**A**) Whole-body insulin levels remained unchanged in the GRKO fish compared to WT. (**B**) The transcript levels of the insulin receptor genes in the brain remained unchanged in GRKO fish compared to WT. (**C**) Loss of GR significantly decreased the ratio of phosphorylated to total Akt (M.W. 60 kDa) in the brain. (**D**) *redd1* mRNA levels significantly decreased in the brain of GRKO fish compared to WT. E. The phosphorylation of mTOR (M.W. 289 kDa) remained unchanged when compared to the WT. Values are means ± SEM (Whole-body insulin n = 6; Protein expression n = 6–9; Transcript abundance n = 6). ns—not significant; *(*P* < 0.05); **(*P* < 0.01).

Dot-blot analysis of whole-body insulin expression showed that insulin expression did not significantly change in the GRKO fish compared to the WT (Fig. 9A; *P* = 0.639). Brain transcript abundance of the insulin receptors, *insra-1* (*P* = 0.07), *insra-2* (*P* = 0.114), *insrb-1* (*P* = 0.195) and *insrb-2* (*P* = 0.139) were also not significantly different between the GRKO and WT fish (Fig. 9B). The ratio of phosphorylated to total Akt expression in the brain of GRKO fish was significantly lower compared to the WT fish (Fig. 9C; *P* = 0.005). The transcript abundance of *redd1* was significantly lower in the brain of GRKO fish compared to the wildtype (Fig. 9D; *P* = 0.043). However, the phospho-mTOR expression was not significantly different between the two genotypes (Fig. 9E; *P* = 0.181).

## Discussion

Our results demonstrate that chronic cortisol elevation inhibits feeding in zebrafish and this appears independent of the higher glucose levels associated with the cortisol-GR activation. Hyperglycemia suppressed feeding in zebrafish, but the molecular mechanisms for this phenotype was distinct from that seen with hypercortisolemia. For instance, while the phosphorylation of AMPK in the brain was reduced in response to hyperglycemia, there was no change in AMPK phosphorylation with cortisol-induced blood glucose elevation. One possible reason for this may be due to the lower brain glucose uptake capacity in response to GR activation by cortisol. In contrast, cortisol treatment reduced the Akt-mTOR signalling and increased the transcript abundance of *redd1* suggesting an enhanced capacity for protein breakdown in the zebrafish brain. The loss of GR did rescue the cortisol-induced *redd1* transcript abundance and the reduction in mTOR phosphorylation supporting a role for GR activation in affecting protein homeostasis in zebrafish^8^. However, the GRKO fish ate less and showed no changes in AMPK phosphorylation, and we propose this may have to do with the positive energy status, including efficient fuel utilization and higher protein content in fish lacking GR^8^. In the present study, we did not observe any changes in the transcript abundance of feeding-related peptides, and this may have to do with the fact that we used whole brain and not the hypothalamus for the analysis. Despite this limitation related to feeding-related peptides, the brain capacity for protein catabolism was enhanced due to GR activation, and we propose the associated increase in amino acid levels as a driver for the cortisol-mediated appetite regulation in fish.

We first confirmed that hyperglycemia, independent of cortisol response, play a role in the feeding suppression by exposing zebrafish to waterborne glucose^21,22^. Hyperglycemia did not evoke a stress response as cortisol levels remained unchanged. Also, the lack of change or reduction in the transcript abundance of GR-responsive genes, including *pck1* and *redd1* indicates that GR was not activated in response to excess glucose^8,10^. The role of AMPK in the regulation of glucose sensing and food intake^15,35^ is well established in mammals^13,16,36,37^, and the evidence suggests that this sensor plays a conserved role in fish^11,14^. In the present study, hyperglycemia reduced the phosphorylation of AMPK in the brain, and this corresponded with the reduced food intake (Figs. 1D and 2A). Similarly, glucose exposure in the hypothalamus of rainbow trout in vitro also led to an inhibition of AMPK, and correspondingly increased the anorectic potential^14^. We did not observe significant changes in the transcript abundance of GLUTs or the feeding-related peptides in the brain suggesting that the steady state transcript levels are not altered by hyperglycemia. Zebrafish did respond to excess glucose by increasing the whole-body insulin expression, but this did not affect whole brain insulin responsiveness as the transcript abundance of either the insulin receptors or the phosphorylation of Akt and mTOR remained unchanged. This is in agreement with studies showing that teleosts respond to hyperglycemia by increasing insulin production, but the target tissue insulin action is not tightly regulated as in mammals^17,18^. Altogether, hyperglycemia altered the nutrient status of the brain, leading to a reduced phosphorylation of AMPK, and the associated suppression of food intake.

To test whether the cortisol-induced hyperglycemia may also play a role in the reduced food intake^38^, we exposed zebrafish to waterborne cortisol^9^. Hypercortisolemia increased blood glucose levels, and this corresponded with an increased transcript abundance of liver *pck1*, supporting GR activation of gluconeogenesis^3,6^. However, little is known about cortisol’s role in central glucose regulation to suppress feeding in fish^15,39^. In the present study, cortisol exposure did inhibit feeding, but this was not accompanied by changes in the brain glucose regulation. For instance, the brain capacity for glucose uptake and the phosphorylation of AMPK was not altered in the cortisol group. One possibility is that the excess glucose in circulation in response to cortisol exposure may not be taken up by the brain to affect nutrient sensing. A previous study also showed that a crowding stressor in rainbow trout (*Oncorhynchus mykiss*), which led to elevated cortisol levels, suppressed the hypothalamic glucose-sensing response^20^. So, it appears that hypercortisolemia may be impeding the glucose uptake capacity of the brain in fish. This was also observed in mammals, as glucocorticoids inhibited the uptake of glucose in the hypothalamus and hippocampus^7^. In mammals, glucocorticoids impair insulin signalling in the brain^40,41^; however, the central glucose sensing and uptake are thought to be mainly insulin-independent unlike peripheral tissues^42^.

In the present study, the lack of change in whole-body insulin expression, despite elevated glucose levels in the cortisol-treated fish, suggests possible impairment of glucose sensing in peripheral tissues^8,43^. This notion finds support from our recent work that showed a reduction in skeletal muscle glucose uptake in zebrafish lacking GR^8^. While there were no changes in the transcript abundance of brain insulin receptors, the reduction in the brain phosphorylation of Akt and mTOR points to a lower capacity for protein synthesis due to hypercortisolemia. In teleosts, insulin appears to be a major regulator of protein metabolism^44^ rather than peripheral glucose regulation^17,18^. The lack of any observable change in the 2-NBDG uptake with insulin either in the control or cortisol-treated fish (Fig. S3) supports a lack of insulin response to brain glucose regulation^17,18^. Consequently, the reduced Akt-mTOR signalling in response to hypercortisolemia observed in the present study may reduce the protein synthetic capacity in the brain of fish. The concomitant upregulation of *redd1*, a GR-responsive gene and a key marker of proteolysis^8,23,25^, suggests an enhanced potential for protein breakdown in the brain due to hypercortisolemia.

The upregulation of *redd1* also suppresses mTOR signalling, thereby inhibiting protein synthesis^23^. Indeed, glucocorticoid-induced inhibition of protein synthesis in skeletal muscle was shown to be regulated by *redd1* suppression of mTORC1^45^. Also, we showed previously that upregulation of *redd1* by cortisol stimulation correspond with increased muscle wasting in zebrafish^8^. Consequently, the upregulation of *redd1* and the suppression of mTOR by chronic cortisol stimulation in the zebrafish brain points to a disruption in protein homeostasis, with a preponderance for protein breakdown. This suggests that other nutrients, apart from glucose, including amino acids may be involved in restricting food intake^11^. In support, a recent study underscored the central amino acid sensing as a key component in the regulation of food intake in fish^46^. In the present study, the chronic cortisol-mediated suppression of feeding was not accompanied by changes in the transcript abundance of key feeding-related peptide (Fig. S2). This is not surprising, as results from fish stress studies have been ambiguous with respect to feeding-related peptide changes, and may underlie the experimental disparities, including the species tested, the type and intensity of the stressor used, the dose and duration of cortisol exposure, and the brain regions used for assessing gene expression^20,38,47–50^.

To investigate if the cortisol-mediated suppression of food intake was due to the activation of GR, we utilized zebrafish lacking GR^9^. The GRKO fish are hypercortisolemic, but the blood glucose levels remain unchanged, likely due to the inability to upregulate gluconeogenesis^8,9,51–54^. In the present study, the GRKO fish ate less food compared to the wildtype, which is in agreement to our previous observation^8^. As the GRKO fish has a functional MR, it is possible that this receptor activation may be playing a role in the reduced food intake. However, the MRKO fish, which has a functional GR, also showed reduced feeding but only in the cortisol treated group^10,24^, supporting a role of GR activation in the appetite regulation.

In fish lacking GR, glucose uptake in the brain was increased, as seen in the muscle^8^, pointing to GR-driven changes in brain fuel utilization. The higher capacity for brain glucose uptake corresponded with alterations in the *slc2a* transcript abundance in the GRKO fish. For instance, the transcript abundance of *slc2a1a, slc2a1b and slc2a2* were downregulated, while the *slc2a3a* and *slc2a12* remained unchanged in the GRKO fish. In rats, chronic hyperglycemia led to a decrease in GLUT1 and GLUT3 expression in the brain^55,56^, suggesting that GLUTs play an important role in the brain glucose regulation, and may be regulated by GR activation. Indeed, studies in zebrafish and rainbow trout (*Oncorhynchus mykiss*) proposed that *slc2a2* may be involved in brain glucose sensing, as it plays an essential role in brain development by facilitating glucose uptake and availability^57–59^.

In the GRKO fish, unlike the fish treated with glucose, the increased capacity for glucose uptake in the brain did not correspond with a reduction in the phosphorylation of AMPK. While the reason for this is unclear, it may have to do with the positive energy status, given the enhanced capacity for brain glucose uptake and protein synthesis. This notion is supported by the higher brain mass and protein content in the GRKO fish in the present study. Also, a faster growth and a higher capacity for protein synthesis and lipid accumulation in the GRKO fish underscores an efficient energy utilization^8,24^, leading to a lower food intake. As *redd1* regulates mTOR^23,25,60^, the reduced transcript abundance of *redd1* in the GRKO fish may also favour mTOR phosphorylation and higher protein synthetic capacity. Consequently, the suppression of mTOR signalling by GR either directly and/or indirectly via *redd1* regulation may play a role in cortisol-mediated protein synthesis inhibition.

**Figure 10:**
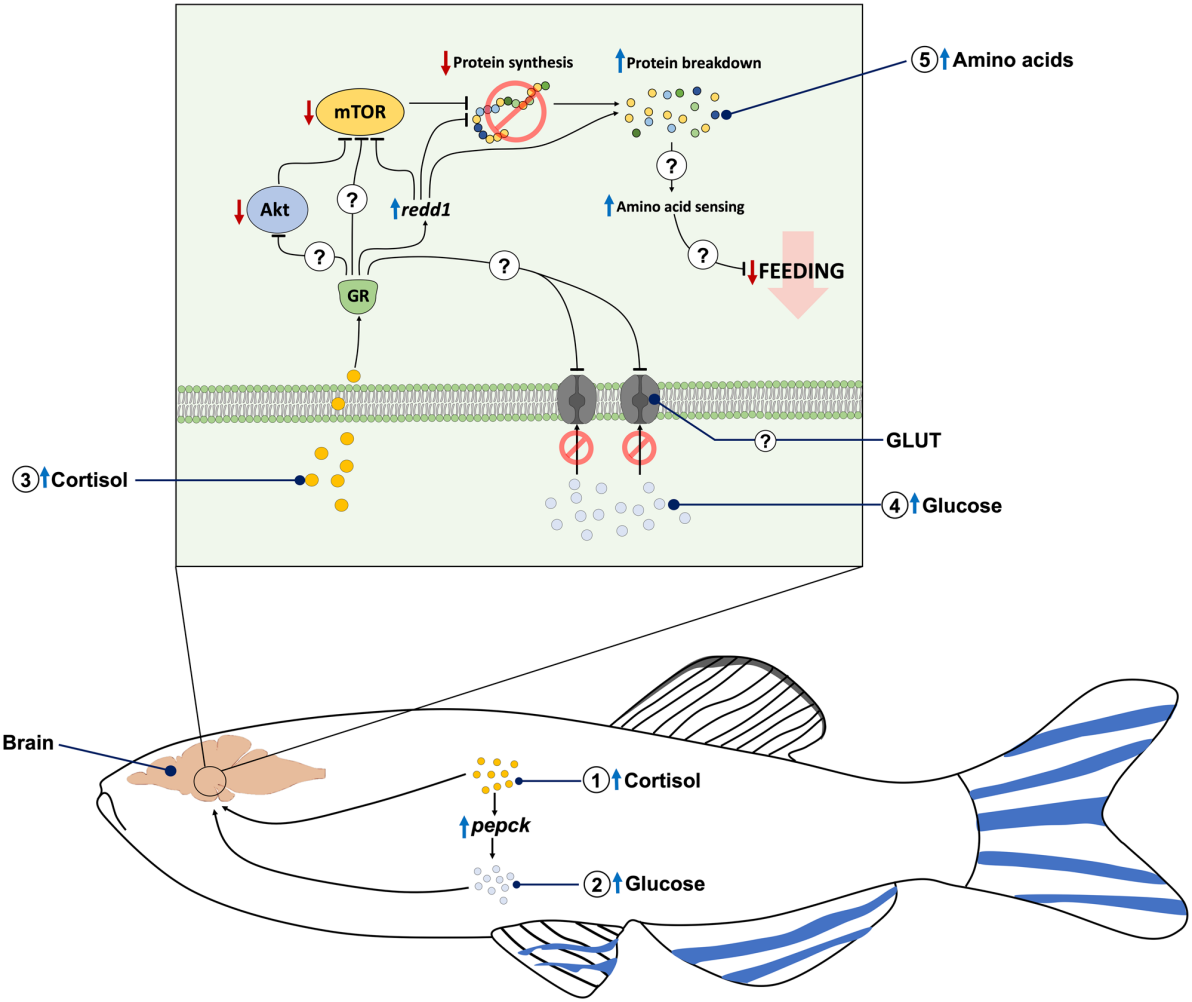
Schematic representation of cortisol action on the regulation of feeding in zebrafish. Hypercortisolemia increased liver *pepck* transcript abundance suggesting enhanced capacity for gluconeogenesis (1), leading to elevated blood glucose levels (2). The elevated cortisol levels activate the glucocorticoid receptor (GR) in the brain (3), which restricts brain glucose uptake (4). GR activation increased *redd1* transcript abundance and decreased the Akt-mTOR signaling, suggesting enhanced capacity for protein catabolism in the brain. We propose that increased protein breakdown may elevate the amino acids pool in the brain and alter the central amino acid sensing (5), which plays a role in the feeding/appetite suppression. “?” denotes mechanism unknown.

In conclusion, high cortisol levels, and stress-independent hyperglycemia, reduced feeding in zebrafish but through distinct mechanisms (See Fig. 10). Despite a hyperglycemic phenotype in both instances, GR activation by cortisol restricted brain glucose uptake and did not alter the phosphorylation of AMPK, unlike stress-independent hyperglycemia, which reduced phosphorylation of this nutrient sensor. The lack of change in AMPK due to cortisol may be associated with an enhanced capacity for brain proteolysis, and the associated increase in amino acid levels. The cortisol-driven changes, including upregulation of *redd1* and the suppression of mTOR phosphorylation were absent in the GRKO fish, pointing to a key role for this receptor activation in affecting brain protein homeostasis. Altogether, cortisol-induced restriction in food intake is driven by GR activation, and we propose that changes in central amino acid sensing may be a possible mechanism.

## Supplementary Information

Supplementary Information.

## Data Availability

The datasets generated during and/or analysed during the current study are available from the corresponding author on reasonable request.

## References

[1] Conde-Sieira M, Chivite M, Míguez JM, Soengas JL (2018). Stress effects on the mechanisms regulating appetite in teleost fish. Front. Endocrinol..

[2] Wendelaar Bonga SE (1997). The stress response in fish. Physiol. Rev..

[3] Faught, E., Aluru, N. & Vijayan, M. M. 4 - The Molecular Stress Response. in *Fish Physiology* (eds. Schreck, C. B., Tort, L., Farrell, A. P. & Brauner, C. J.) vol. 35 113–166 (Academic Press, 2016).

[4] Charmandari E, Tsigos C, Chrousos G (2005). Endocrinology of the stress response. Annu. Rev. Physiol..

[5] Mommsen TP, Vijayan MM, Moon TW (1999). Cortisol in teleosts: Dynamics, mechanisms of action, and metabolic regulation. Rev. Fish Biol. Fish..

[6] Faught E, Vijayan MM (2016). Mechanisms of cortisol action in fish hepatocytes. Comp. Biochem. Physiol. B Biochem. Mol. Biol..

[7] Kuo T, McQueen A, Chen T-C, Wang J-C (2015). Regulation of glucose homeostasis by glucocorticoids. Adv. Exp. Med. Biol..

[8] Faught E, Vijayan MM (2019). Loss of the glucocorticoid receptor in zebrafish improves muscle glucose availability and increases growth. Am. J. Physiol. Endocrinol. Metab..

[9] Faught, E. & Vijayan, M. M. The mineralocorticoid receptor is essential for stress axis regulation in zebrafish larvae. *Sci. Rep.* **8**, (2018). 10.1038/s41598-018-36681-w PMC6308233 30591705

[10] Faught E, Vijayan MM (2020). Glucocorticoid and mineralocorticoid receptor activation modulates postnatal growth. J. Endocrinol..

[11] Conde-Sieira M, Soengas JL (2017). Nutrient sensing systems in fish: Impact on food intake regulation and energy homeostasis. Front. Neurosci..

[12] Lane MD, Cha SH (2009). Effect of glucose and fructose on food intake via malonyl-CoA signaling in the brain. Biochem. Biophys. Res. Commun..

[13] Minokoshi Y (2004). AMP-kinase regulates food intake by responding to hormonal and nutrient signals in the hypothalamus. Nature.

[14] Otero-Rodiño C (2017). Changes in the levels and phosphorylation status of Akt, AMPK, CREB and FoxO1 in hypothalamus of rainbow trout under conditions of enhanced glucosensing activity. J. Exp. Biol..

[15] Soengas JL (2021). Integration of nutrient sensing in fish hypothalamus. Front. Neurosci..

[16] Zhang J (2015). ERK1/2 mediates glucose-regulated POMC gene expression in hypothalamic neurons. J. Mol. Endocrinol..

[17] Moon TW (2001). Glucose intolerance in teleost fish: Fact or fiction?. Comp. Biochem. Physiol. B Biochem. Mol. Biol..

[18] Polakof S, Panserat S, Soengas JL, Moon TW (2012). Glucose metabolism in fish: A review. J. Comp. Physiol. B.

[19] Maddison LA, Joest KE, Kammeyer RM, Chen W (2015). Skeletal muscle insulin resistance in zebrafish induces alterations in β-cell number and glucose tolerance in an age- and diet-dependent manner. Am. J. Physiol. Endocrinol. Metab..

[20] Conde-Sieira M (2010). Effect of different glycaemic conditions on gene expression of neuropeptides involved in control of food intake in rainbow trout; interaction with stress. J. Exp. Biol..

[21] Gleeson M, Connaughton V, Arneson LS (2007). Induction of hyperglycaemia in zebrafish (*Danio rerio*) leads to morphological changes in the retina. Acta Diabetol..

[22] Capiotti KM (2014). Persistent impaired glucose metabolism in a zebrafish hyperglycemia model. Comp. Biochem. Physiol. B Biochem. Mol. Biol..

[23] Britto FA, Dumas K, Giorgetti-Peraldi S, Ollendorff V, Favier FB (2020). Is REDD1 a metabolic double agent? Lessons from physiology and pathology. Am. J. Physiol. Cell Physiol..

[24] Faught E, Vijayan MM (2019). Postnatal triglyceride accumulation is regulated by mineralocorticoid receptor activation under basal and stress conditions. J. Physiol..

[25] Gordon BS, Steiner JL, Williamson DL, Lang CH, Kimball SR (2016). Emerging role for regulated in development and DNA damage 1 (REDD1) in the regulation of skeletal muscle metabolism. Am. J. Physiol.-Endocrinol. Metab..

[26] Faught E, Best C, Vijayan MM (2016). Maternal stress-associated cortisol stimulation may protect embryos from cortisol excess in zebrafish. R. Soc. Open Sci..

[27] Eames SC, Philipson LH, Prince VE, Kinkel MD (2010). Blood sugar measurement in zebrafish reveals dynamics of glucose homeostasis. Zebrafish.

[28] Ciarlo C (2017). A chemical screen in zebrafish embryonic cells establishes that Akt activation is required for neural crest development. Elife.

[29] Collodet C (2019). AMPK promotes induction of the tumor suppressor FLCN through activation of TFEB independently of mTOR. FASEB J..

[30] Slade L, Cowie A, Martyniuk CJ, Kienesberger PC, Pulinilkunnil T (2017). Dieldrin augments mTOR signaling and regulates genes associated with cardiovascular disease in the adult zebrafish heart (*Danio rerio*). J. Pharmacol. Exp. Ther..

[31] Kimmel RA (2015). Diabetic pdx1-mutant zebrafish show conserved responses to nutrient overload and anti-glycemic treatment. Sci. Rep..

[32] Livak KJ, Schmittgen TD (2001). Analysis of relative gene expression data using real-time quantitative PCR and the 2−ΔΔCT method. Methods.

[33] Zhang X, Yang S, Chen J, Su Z (2019). Unraveling the regulation of hepatic gluconeogenesis. Front. Endocrinol..

[34] Ota KT (2014). REDD1 is essential for stress-induced synaptic loss and depressive behavior. Nat. Med..

[35] Ronnett GV, Ramamurthy S, Kleman AM, Landree LE, Aja S (2009). AMPK in the brain: Its roles in energy balance and neuroprotection. J. Neurochem..

[36] Claret M (2007). AMPK is essential for energy homeostasis regulation and glucose sensing by POMC and AgRP neurons. J. Clin. Invest..

[37] Oh TS, Cho H, Cho JH, Yu S-W, Kim E-K (2016). Hypothalamic AMPK-induced autophagy increases food intake by regulating NPY and POMC expression. Autophagy.

[38] Bernier NJ, Bedard N, Peter RE (2004). Effects of cortisol on food intake, growth, and forebrain neuropeptide Y and corticotropin-releasing factor gene expression in goldfish. Gen. Comp. Endocrinol..

[39] Delgado MJ, Cerdá-Reverter JM, Soengas JL (2017). Hypothalamic integration of metabolic, endocrine, and circadian signals in fish: Involvement in the control of food intake. Front. Neurosci..

[40] Piroli GG (2007). Corticosterone impairs insulin-stimulated translocation of GLUT4 in the rat hippocampus. Neuroendocrinology.

[41] Steiner JL, Bardgett ME, Wolfgang L, Lang CH, Stocker SD (2014). Glucocorticoids attenuate the central sympathoexcitatory actions of insulin. J. Neurophysiol..

[42] Agrawal R (2021). Insulin action in the brain regulates both central and peripheral functions. Am. J. Physiol. Endocrinol. Metab..

[43] Fichna M, Fichna P (2017). Glucocorticoids and beta-cell function. Endokrynol. Pol..

[44] Mommsen T, Plisetskaya E (1991). Insulin in fishes and Agnathans - History, structure, and metabolic-regulation. Rev. Aquat. Sci..

[45] Britto FA (2014). REDD1 deletion prevents dexamethasone-induced skeletal muscle atrophy. Am. J. Physiol.-Endocrinol. Metab..

[46] Comesaña S (2018). Evidence for the presence in rainbow trout brain of amino acid-sensing systems involved in the control of food intake. Am. J. Physiol. Regul. Integr. Comp. Physiol..

[47] Janzen WJ, Duncan CA, Riley LG (2012). Cortisol treatment reduces ghrelin signaling and food intake in tilapia, *Oreochromis mossambicus*. Domest. Anim. Endocrinol..

[48] Upton KR, Riley LG (2013). Acute stress inhibits food intake and alters ghrelin signaling in the brain of tilapia (*Oreochromis mossambicus*). Domest. Anim. Endocrinol..

[49] Cortés R (2018). Effects of acute handling stress on short-term central expression of orexigenic/anorexigenic genes in zebrafish. Fish Physiol. Biochem..

[50] Naderi F (2018). Involvement of cortisol and sirtuin1 during the response to stress of hypothalamic circadian system and food intake-related peptides in rainbow trout, *Oncorhynchus mykiss*. Chronobiol. Int..

[51] Griffiths BB (2012). A zebrafish model of glucocorticoid resistance shows serotonergic modulation of the stress response. Front. Behav. Neurosci..

[52] Ziv L (2013). An affective disorder in zebrafish with mutation of the glucocorticoid receptor. Mol. Psychiatry.

[53] Facchinello N (2017). nr3c1 null mutant zebrafish are viable and reveal DNA-binding-independent activities of the glucocorticoid receptor. Sci. Rep..

[54] Faught E, Santos HB, Vijayan MM (2020). Loss of the glucocorticoid receptor causes accelerated ovarian ageing in zebrafish. Proc. R. Soc. B Biol. Sci..

[55] Hou W (2007). Influence of blood glucose on the expression of glucose transporter proteins 1 and 3 in the brain of diabetic rats. Chin. Med. J. (Engl.).

[56] Lutz AJ, Pardridge WM (1993). Insulin therapy normalizes GLUT1 glucose transporter mRNA but not immunoreactive transporter protein in streptozocin-diabetic rats. Metabolism.

[57] Marín-Juez R (2015). GLUT2-mediated glucose uptake and availability are required for embryonic brain development in zebrafish. J. Cereb. Blood Flow Metab..

[58] Polakof S, Míguez JM, Moon TW, Soengas JL (2007). Evidence for the presence of a glucosensor in hypothalamus, hindbrain, and Brockmann bodies of rainbow trout. Am. J. Physiol.-Regul. Integr. Comp. Physiol..

[59] Polakof S, Míguez JM, Soengas JL (2008). Dietary carbohydrates induce changes in glucosensing capacity and food intake of rainbow trout. Am. J. Physiol. Regul. Integr. Comp. Physiol..

[60] Katiyar S (2009). REDD1, an inhibitor of mTOR signalling, is regulated by the CUL4A–DDB1 ubiquitin ligase. EMBO Rep..

