# Supplementary material for: Glucocorticoid receptor activation reduces food intake independent of hyperglycemia in zebrafish
Source: Sci Rep. 2022 Sep 20;12:15677. doi: 10.1038/s41598-022-19572-z (PMC9489701; doi:10.1038/s41598-022-19572-z)
Supplement: Supplementary file 1 — Supplementary Information. [file 41598_2022_19572_MOESM1_ESM.pdf]

Supplemental figures

Figure S1: The effect of glucose treatment on expression of genes related to central appetite regulation. Whole-brain transcript levels of *npy* (A), *crh* (B), *pomca* (C), *mc3r* (D), *mc4r* (E) and *leptin a* (*lepa*) (F) did not change when compared to control. Values are means ± SEM (n=6), ns; not significant.

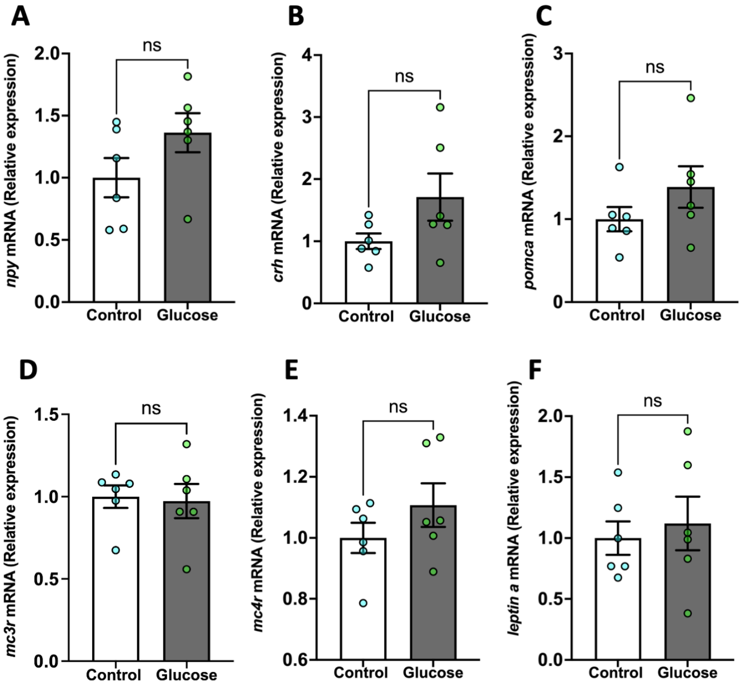

Figure S2: The effect of cortisol exposure on expression of genes related to central appetite regulation. Whole-brain transcript levels of *npy* (A), *crh* (B), *pomca* (C), *mc3r* (D), *mc4r* (E) and *leptin a* (*lepa*) (F) did not change when compared to control. Values are means ± SEM (n=5-6), ns; not significant.

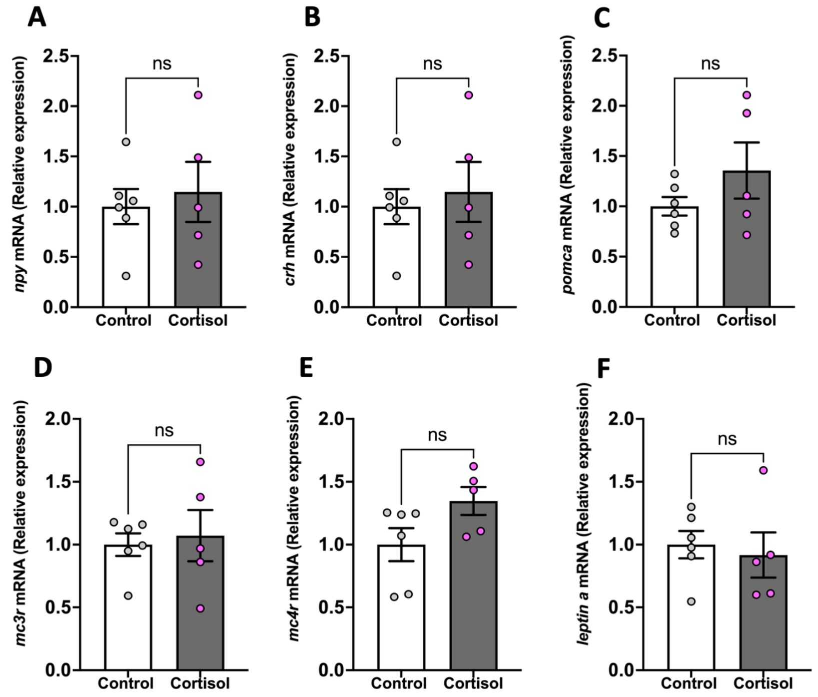

Figure S3: Insulin does not affect brain glucose uptake. A. Insulin injection did not significantly change brain glucose uptake in both the control and cortisol treated group compared to the saline injected fish. Values are means ± SEM (RFU n=5-6), ns; not significant. RFU, relative fluorescent units.

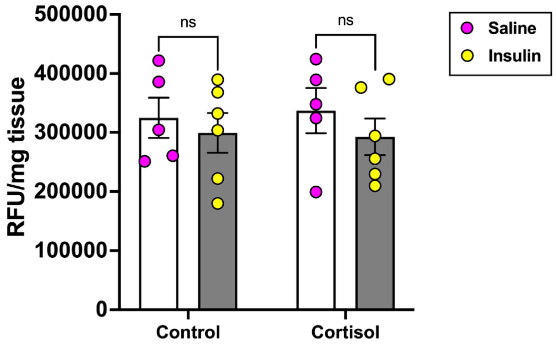

Figure S4: GRKO fish were hypercortisolemic. A. Whole-body cortisol levels were higher in the GRKO fish compared to the WT. B. Plasma glucose levels did not change in the GRKO fish compared to WT. C. The expression of *pck1* in the liver remained unchanged in the GRKO fish compared to the control. Values are means ± SEM (Whole-body cortisol n=6-8; Plasma glucose n=12-14; Transcripts n=6). *Significantly different from the WT, ns; not significant (*t*-test; *$P <$ 0.05).

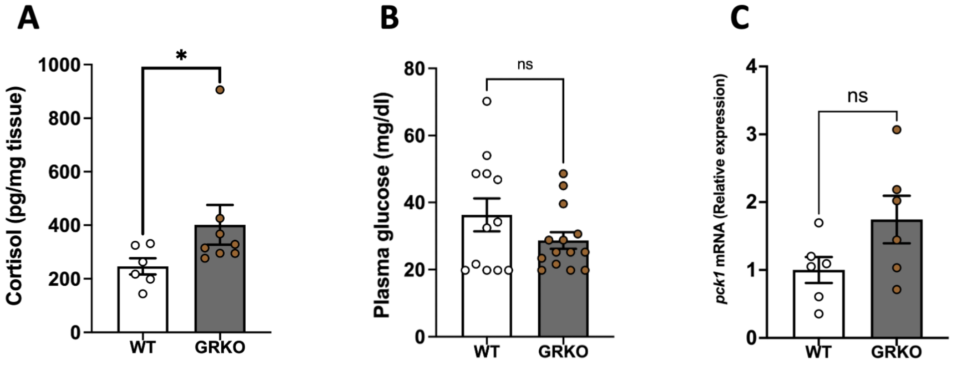

Figure S5: Loss of GR affects genes related to central appetite regulation. Whole-brain transcript levels of *leptin a (lepa)* (F) significantly decreased in the GRKO fish, but the expression of *npy* (A), *crh* (B), *pomca* (C), *mc3r* (D) and *mc4r* (E) did not change when compared to WT. Values are means ± SEM (n=5-6) *Significantly different from WT, ns; not significant (*t*-test; *$P < 0.05$).

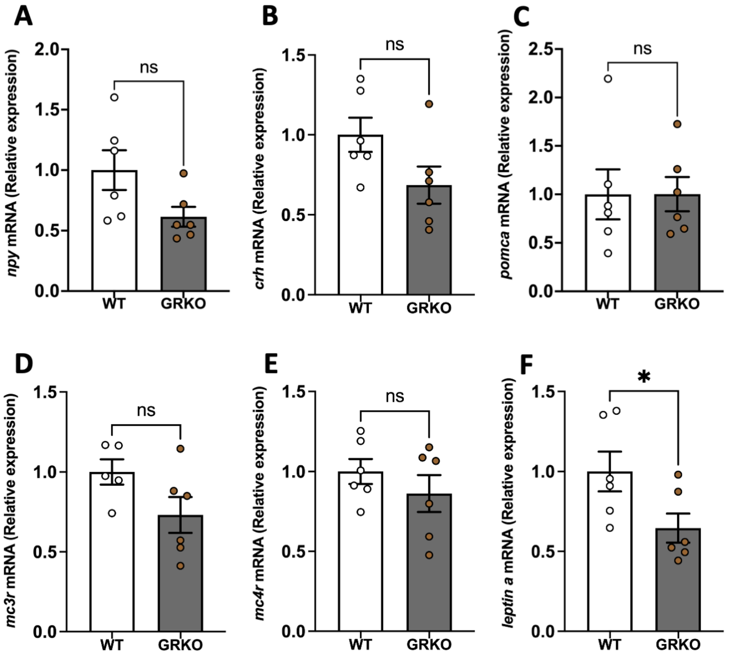

Figure S6: Quantification of AMPK upper band and doublet. Glucose treatment significantly decreased the ratio of phosphorylated to total AMPK (M.W.:62 kDa) in the upper band (A), and doublet (D) in the brain compared to the control. Cortisol treatment did not change the ratio of phosphorylated to total AMPK (M.W.:62 kDa) in the upper band (B) or doublet (E) in the brain compared to the control. The ratio of phosphorylated to total AMPK (M.W.:62 kDa) in the upper band (C), and doublet (F) remained unchanged in the GRKO fish compared to WT. Values are means ± SEM (n=4-6) *Significantly different from control, ns; not significant (*t*-test; *$P < 0.05$, ** $P < 0.01$).

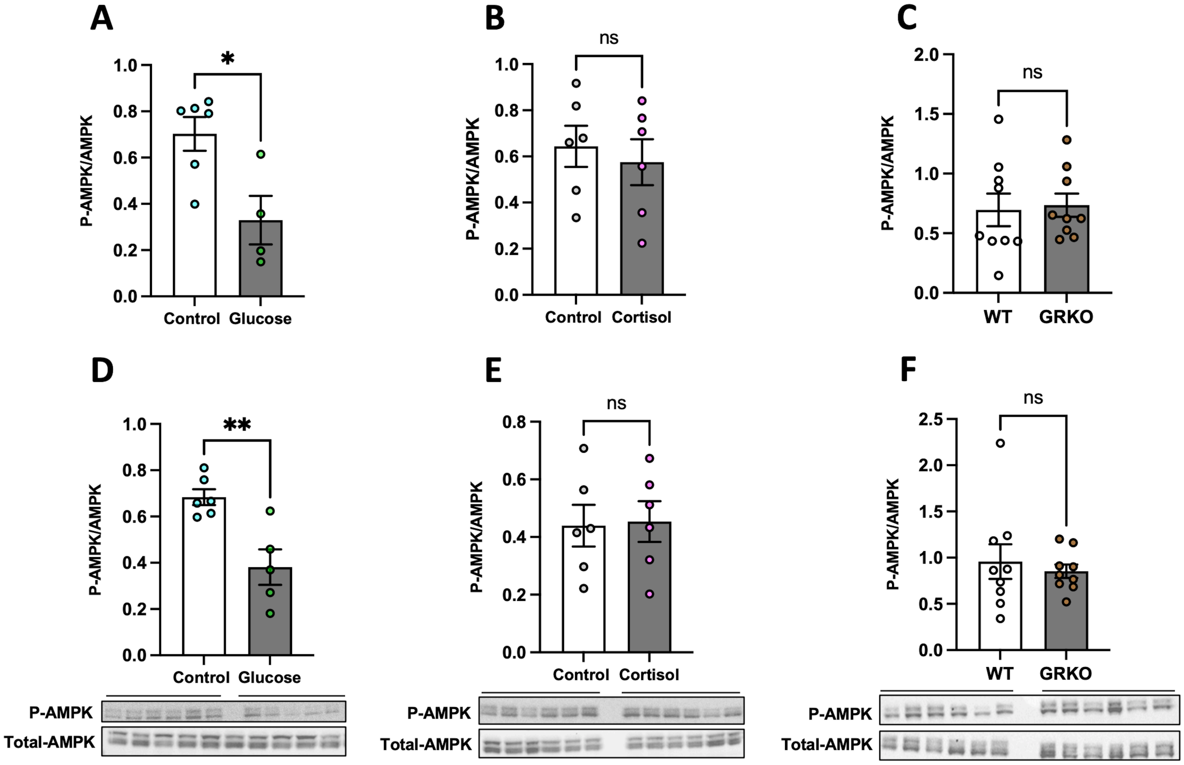

Table S1: Primers used for quantitative real-time PCR.

Gene	Tm	Forward sequence	Reverse sequence	Genbank Accession No.	Ref.
pck1	60	5'-AGTGGGACAAAGCCATGAAC-3'	5'-TCAGCTCCACCCCTATCTTG-3'	NM_214751	[1]
β-actin	60	5'-TGTCCCTGTATGCCTCTGGT-3'	5'-AAGTCCAGACGGAGGATG-3'	NM_181601	[2]
crf/crh	60	5'-CACCGCCGTATGAATGTAGA-3'	5'-GAAGTACTCCTCCCCCAAGC-3'	NM_001007379	[3]
mc3r	60	5’-CTGATTCTCCAGCAGGGACG-3’	5’-ACCACAGCCGAGATGACAAG-3’	NM_180972	
mc4r	60	5’-AGCCCTCTCATGGTGACAGA-3’	5’-CGATGGCTGCAATCACAAGG-3’	NM_173278	
npy	60	5'-TCAAGCGCTGACACCTTAAT-3'	5'-GATGAGATCACCATGCCAAATG-3'	NM_131074	[4]
pomca	60	5'-GAAGAGGAATCCGCCGAAA-3'	5'-CCAGTGGGTTTAAAGGCATCTC -3'	NM_181438	[5]
slc2a1a	60	5'-TGACCGGCCCATACGTTTTC-3'	5'-ATCATCTCGGTTATATTTATCTGCC-3'	NM_001039808	[6]
slc2a1b	60	5'-CCATTTCTCCTGGGCTTTACCTTTA-3'	5'-CAGATTTGGCTTTGCTTTCCTCGTT-3'	XM_002662528	[6]
slc2a2	60	5'-TTAACAGGCACGCTCGCTCT-3'	5'-TTCATGCTCTGTGCCATTTCC-3'	NM_001042721	[6]
slc2a3a	60	5'-TCGTCAATGTCTTGGCTCTG-3'	5'-CAACATACATTGGCGTGAGG-3'	NM_001002643	[6]
slc2a12	60	5'-GGGACAATCCTGGACCACTA-3'	5'-ACATCCCAACCAGCATTCTC-3'	NM_200538	[6]
insra1	60	5'-CAACATGCCCCCTCACCACT-3'	5'-CGACACACATGTTGTTGTG-3'	NM_001142672	[7]
insra2	60	5'-GGAGCCCCACTCGTCTAACAAA-3'	5'-CGCCGTTGTGAATGACGTATTC-3'	NM_001142672	[7]
insrb1	60	5'-GACTGATTACTATCGCAAGGG-3'	5'-TCCAGGTATCCTCCGTCCAT-3'	NM_001123229	[7]
insrb2	60	5'-CCACCGCCAACCCTAAAGGA-3'	5'-TTGCGATAGTAATCAGTCTCGTAAAT-3'	NM_001123229	[7]
lepa	60	5'-ATCGTCAGAATCAGGGAACAC-3'	5'-CCCAATGATGAGCGTTGGA-3'	NM_001128576	[8]
redd1	60	5'-ATGCAAGATCAGTTGATTTCCAGCC-3'	5'-TCAGCATTCTTCAATCAGGAGCTCT-3'	NM_200107	[9]
gck	60	5'-GACACAGGGGACAGAAAGCA-3'	5'-CCACCCCCACAGTGATCTTT-3'	NM_001045385	[10]

Full images of blots shown in the main manuscript

Figure 2A
P-AMPK blot
Arrow indicates P-AMPK (62kDa)
Lane 1 : Protein ladder
Lane 2-7: Protein bands in the control
Lane 9-13: Protein bands in the glucose group

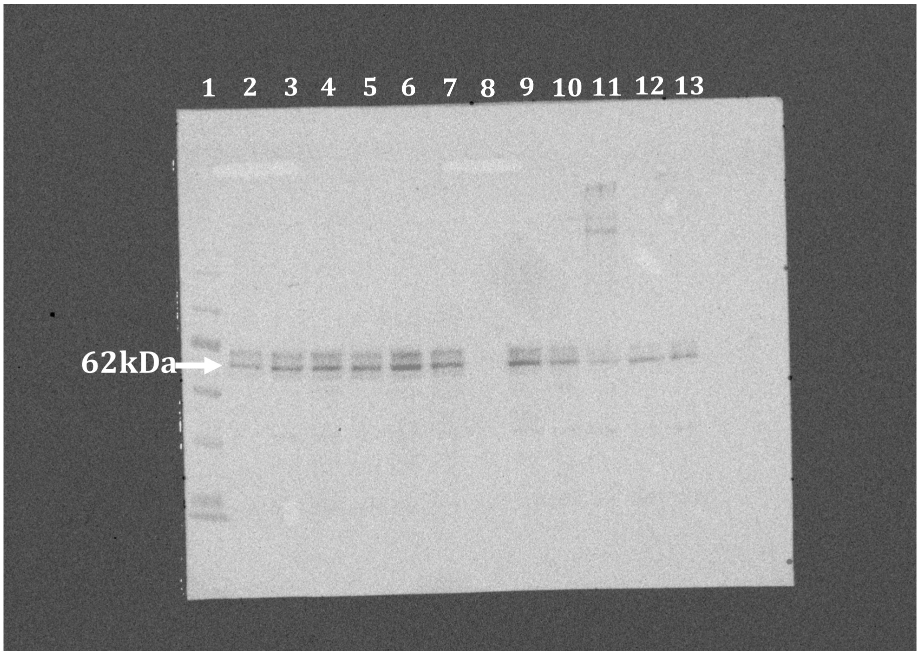

Figure 2A
Total AMPK blot
Arrow indicates Total-AMPK (62kDa)
Lane 1 : Protein ladder
Lane 2-7: Protein bands in the control
Lane 8-13: Protein bands in the glucose group
Lane 15: Rainbow trout brain

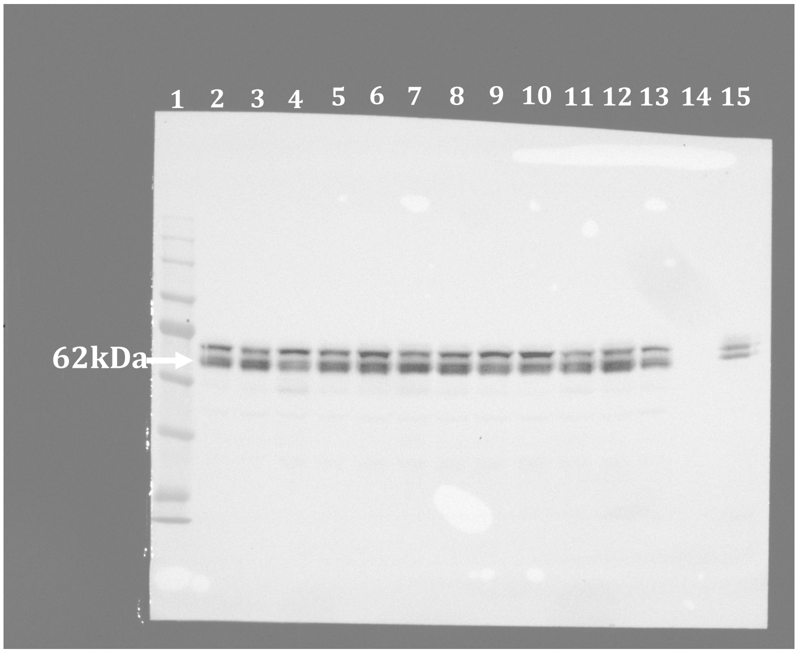

Figure 3B
P-Akt
Arrow indicates P-Akt (60 kDa)
Lane 1: Protein ladder
Lane 2-7: Protein bands in the control
Lane 9-14: Protein bands in the glucose group

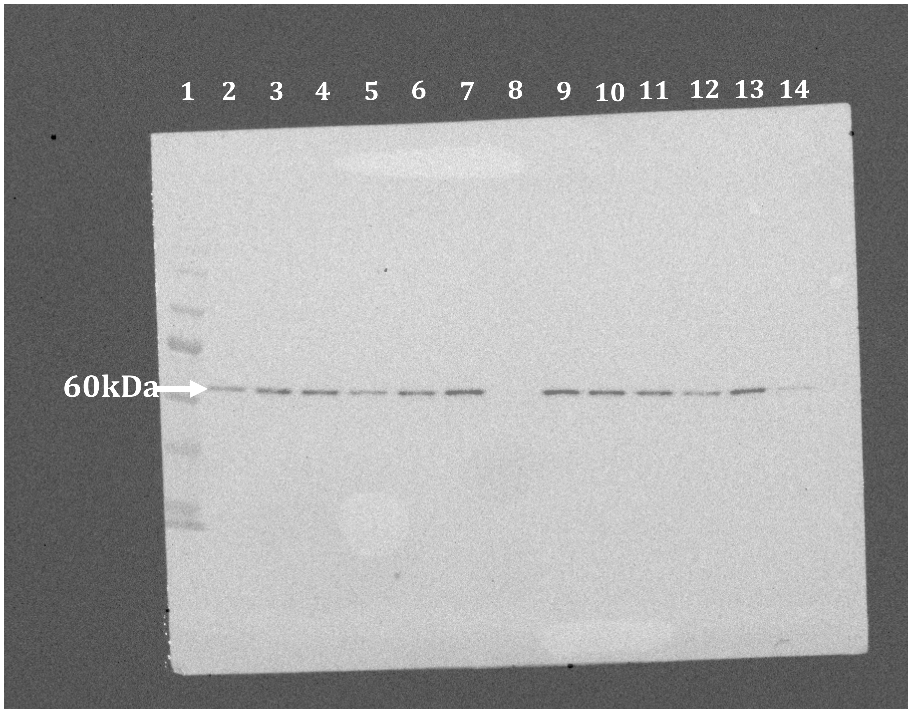

Figure 3B
Total-Akt
Arrow indicates Total-Akt (60 kDa)
Lane 1: Protein ladder
Lane 2-7: Protein bands in the control
Lane 9-14: Protein bands in the glucose group
Lane 15: Rainbow trout brain

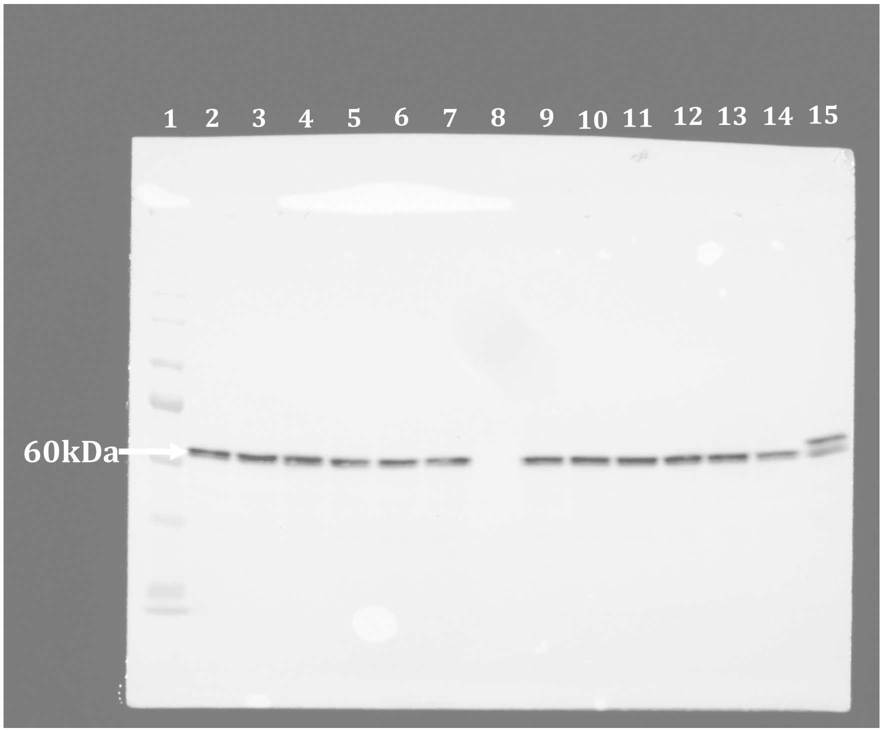

Figure 3C
P-mTOR
Arrow indicates P-mTOR (289 kDa)
Lane 1: Protein ladder
Lane 2-7: Protein bands in the control
Lane 9-14: Protein bands in the glucose group
Lane 15: Rainbow trout brain

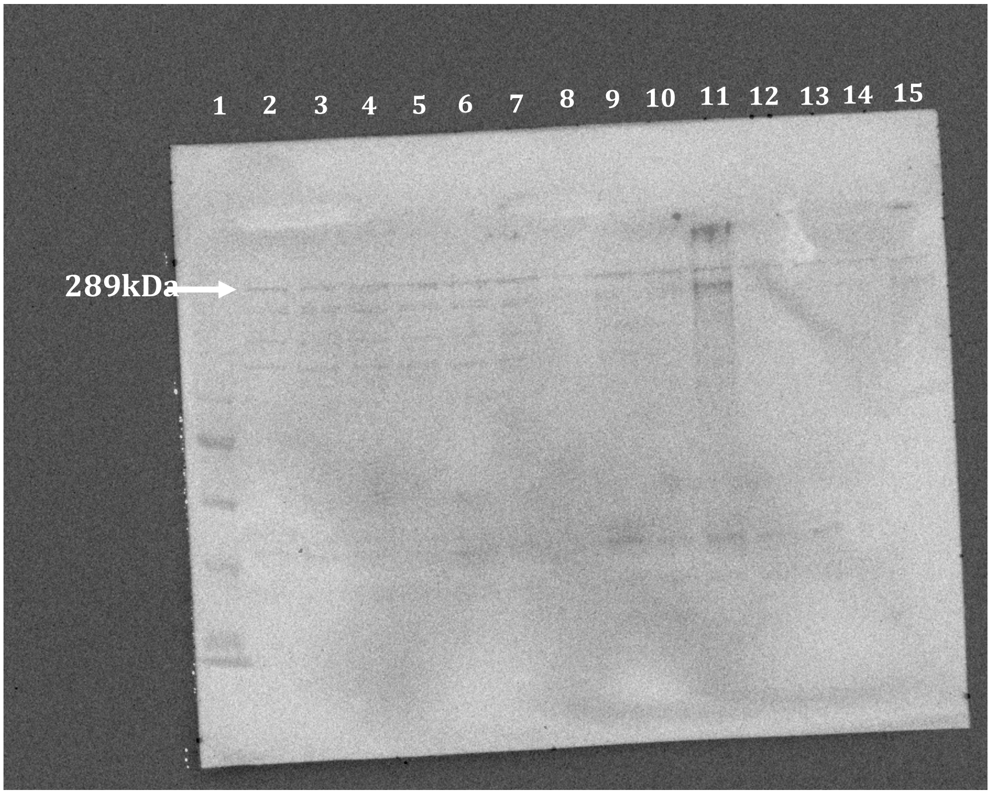

Figure 3C
B-actin
Arrow indicates B-actin (42 kDa)
Lane 1: Protein ladder
Lane 2-7: Protein bands in the control
Lane 9-14: Protein bands in the glucose group
Lane 15: Rainbow trout brain

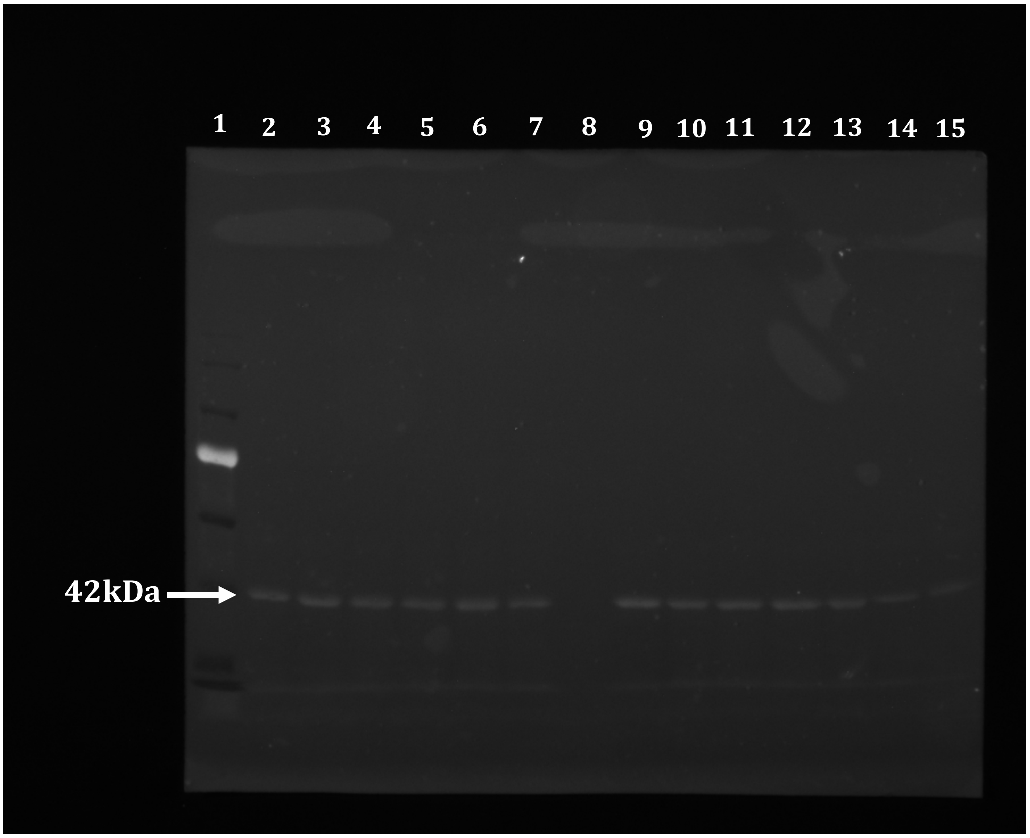

Figure 5C
P-AMPK blot
Arrow indicates P-AMPK (62kDa)
Lane 1: Protein ladder
Lane 2-7: Protein bands in the control
Lane 9-14: Protein bands in the cortisol group

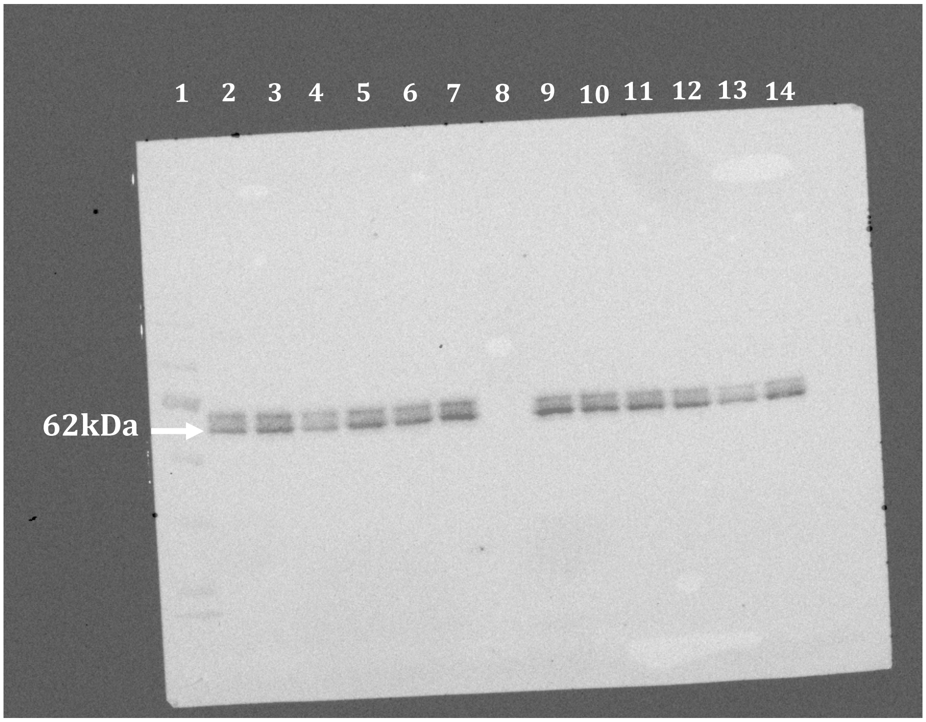

Figure 5C
Total-AMPK blot
Arrow indicates Total-AMPK (62kDa)
Lane 1: Protein ladder
Lane 2-7: Protein bands in the control
Lane 9-14: Protein bands in the cortisol group
Lane 15: Rainbow trout brain

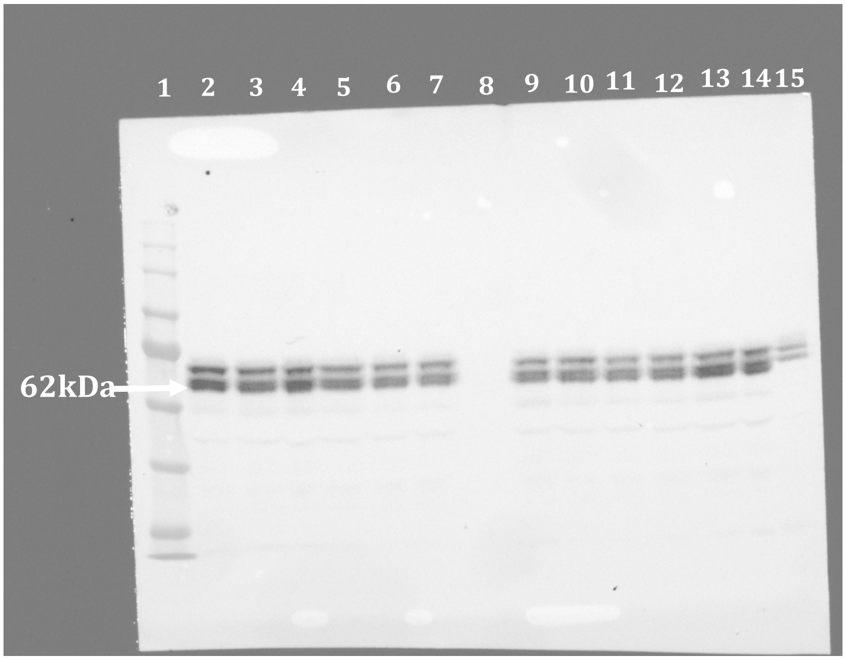

Figure 6C
P-Akt blot
Arrow indicates P-Akt (60kDa)
Lane 1: Protein ladder
Lane 2-7: Protein bands in the control
Lane 9-14: Protein bands in the cortisol group
Lane 15: Rainbow trout brain

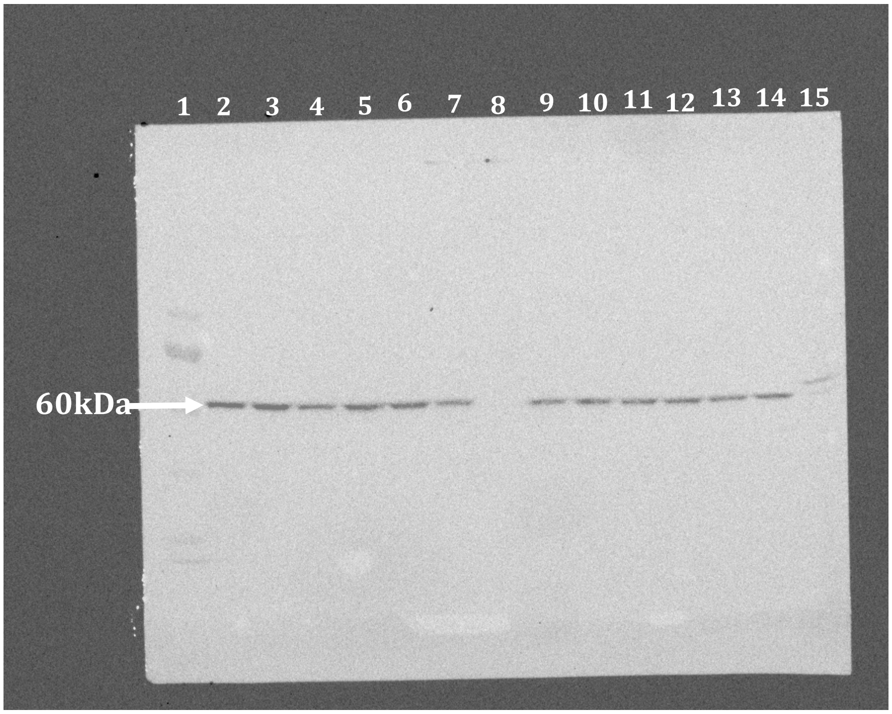

Figure 6C
Total-Akt blot
Arrow indicates Total-Akt (60kDa)
Lane 1: Protein ladder
Lane 2-7: Protein bands in the control
Lane 9-14: Protein bands in the cortisol group
Lane 15: Rainbow trout brain

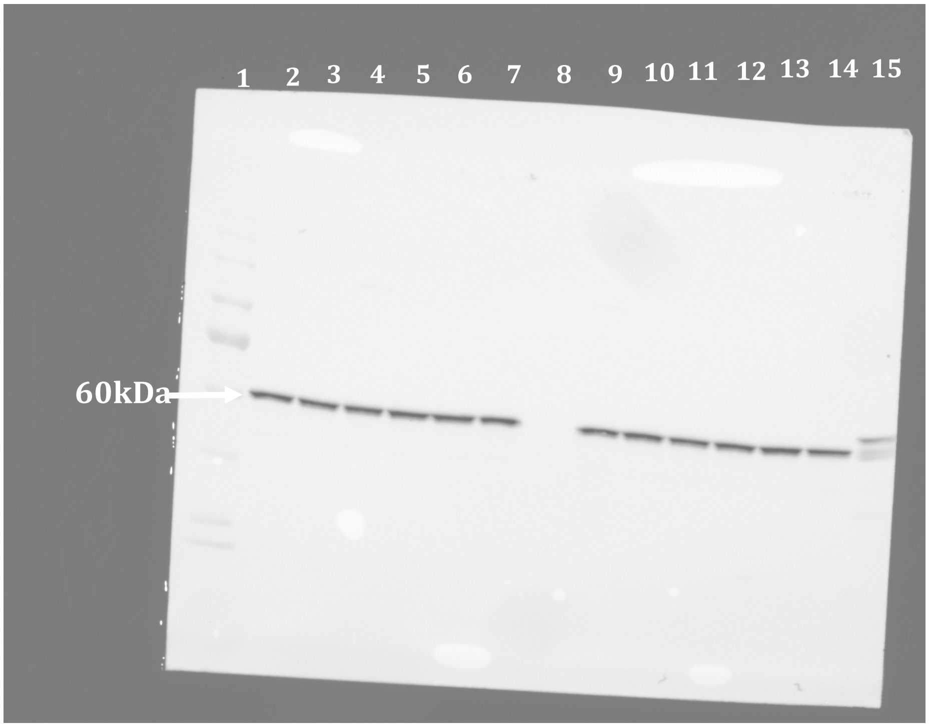

Figure 6D
P-mTOR
Arrow indicates P-mTOR (289 kDa)
Lane 1: Protein ladder
Lane 2-7: Protein bands in the control
Lane 9-14: Protein bands in the cortisol group
Lane 15: Rainbow trout brain

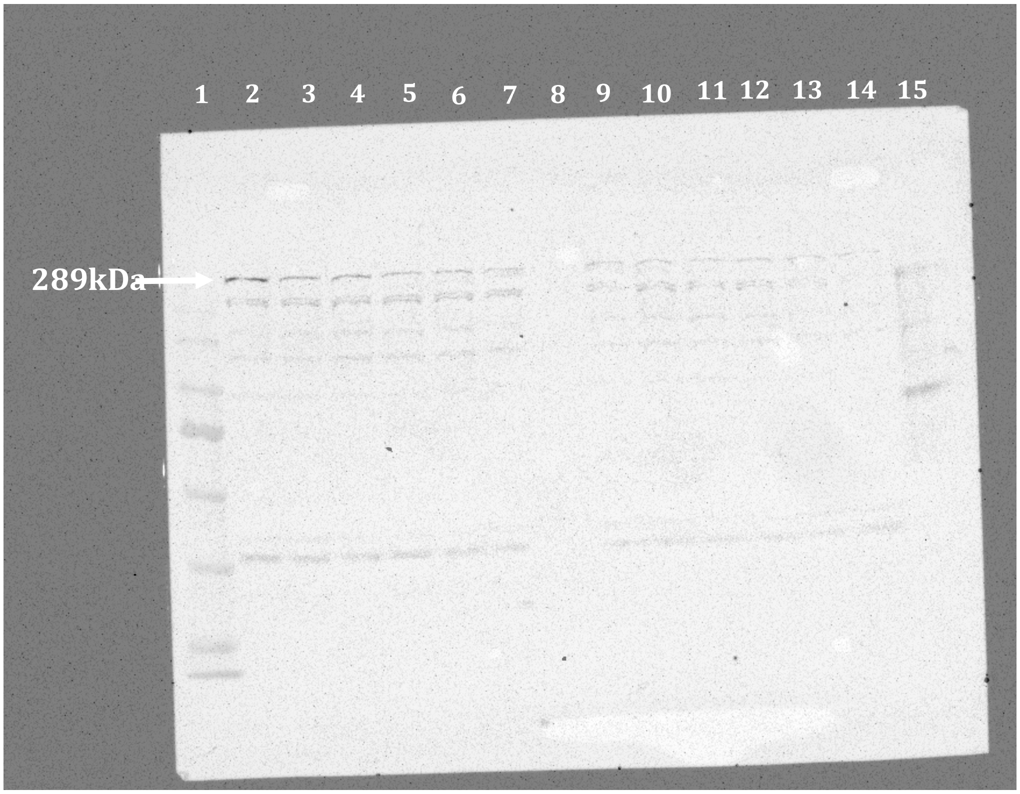

Figure 6D
B-actin
Arrow indicates B-actin (42 kDa)
Lane 1: Protein ladder
Lane 2-7: Protein bands in the control
Lane 9-14: Protein bands in the cortisol group
Lane 15: Rainbow trout brain

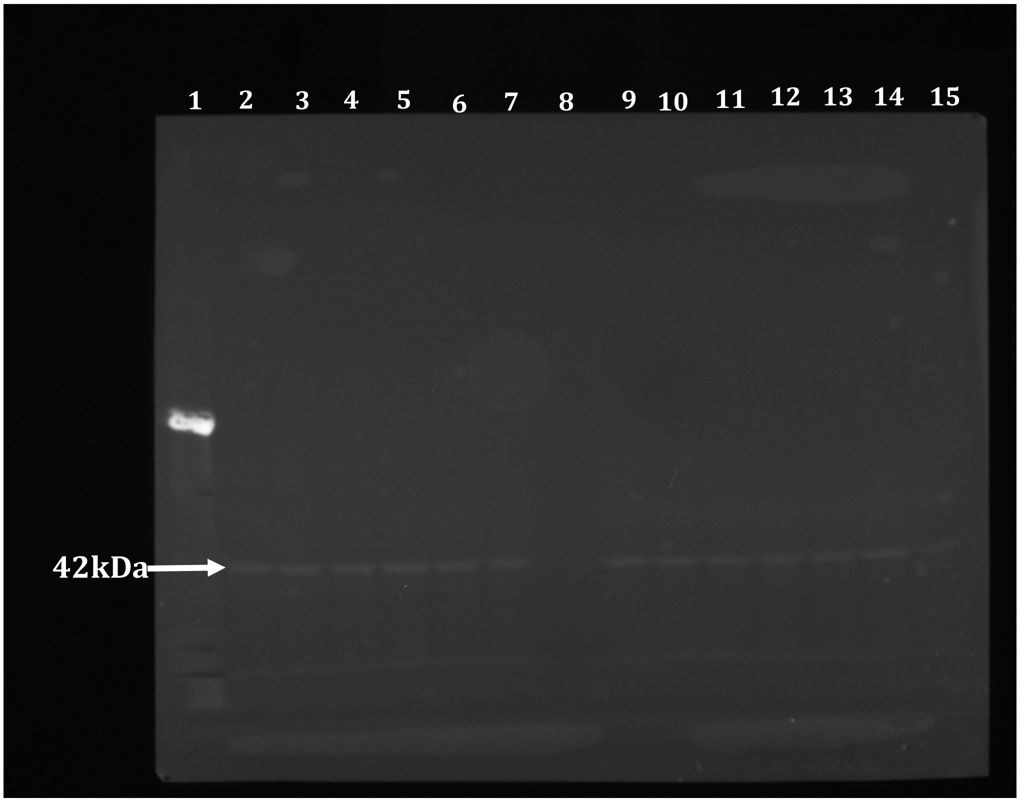

Figure 8C
P-AMPK blot 1
Arrow indicates P-AMPK (62 kDa)
Lane 1: Protein ladder
Lane 2-4: Protein bands in the WT
Lane 6-8: Protein bands in the GRKO

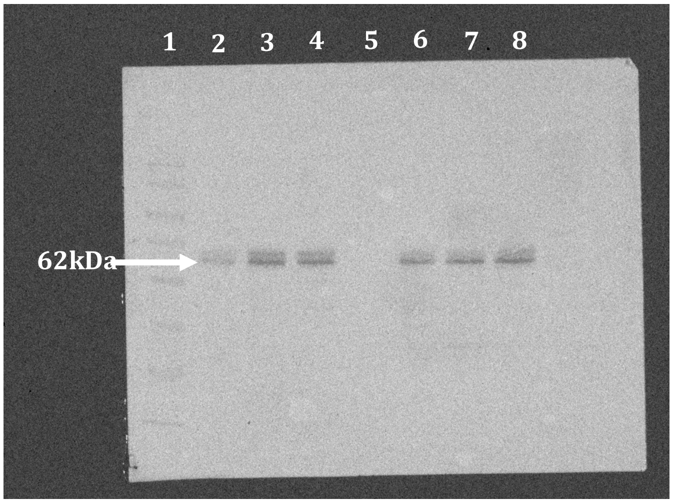

Figure 8C
P-AMPK blot 2
Arrow indicates P-AMPK (62 kDa)
Lane 1: Protein ladder
Lane 2-7: Protein bands in the WT
Lane 9-14: Protein bands in the GRKO

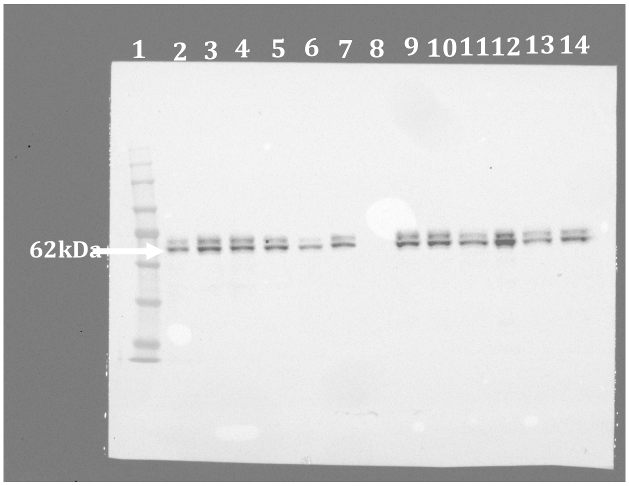

Figure 8C
Total-AMPK blot 1
Arrow indicates Total-AMPK (62 kDa)
Lane 1: Protein ladder
Lane 2-4: Protein bands in the WT
Lane 6-8: Protein bands in the GRKO
Lane 9: Rainbow trout brain

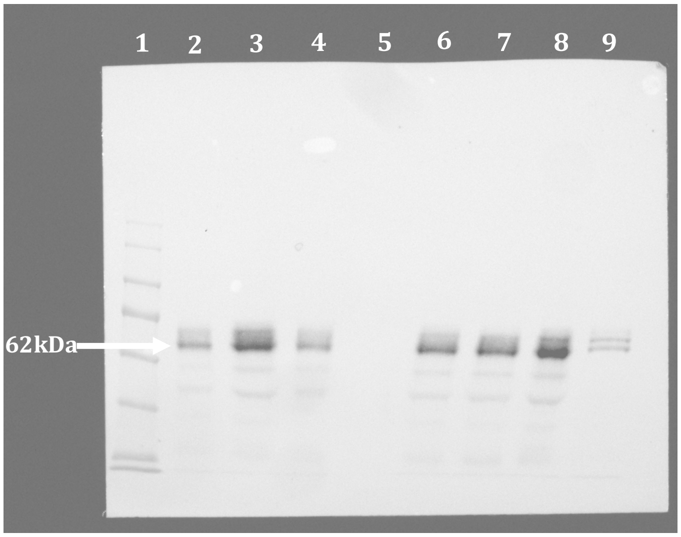

Figure 8C
Total-AMPK blot 2
Arrow indicates Total-AMPK (62 kDa)
Lane 1: Protein ladder
Lane 2-7: Protein bands in the WT
Lane 9-14: Protein bands in the GRKO

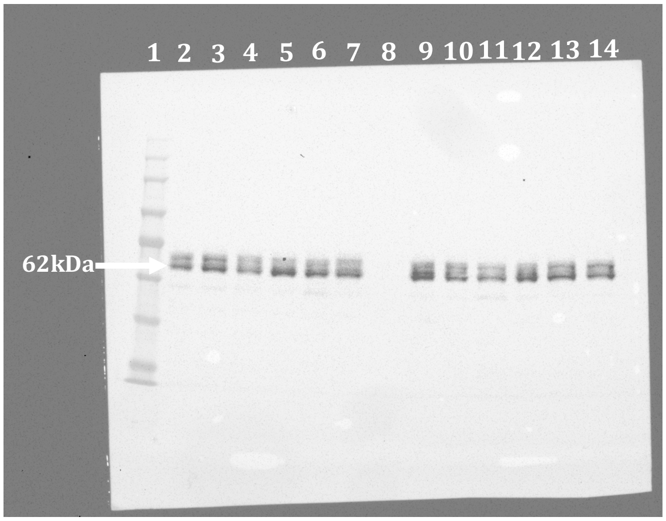

Figure 9C
P-Akt blot 1
Arrow indicates P-Akt (60 kDa)
Lane 1: Rainbow trout brain
Lane 2: Protein ladder
Lane 3-5: Protein bands in the WT
Lane 7-9: Protein bands in the GRKO

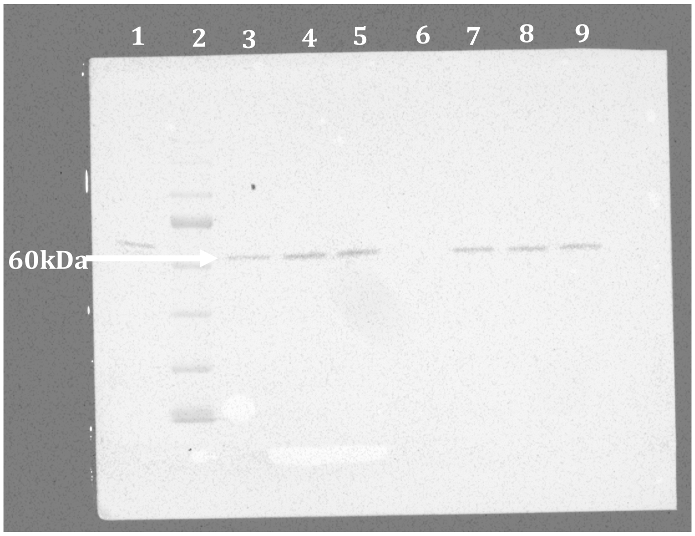

Figure 9C
P-Akt blot 2
Arrow indicates P-Akt (60 kDa)
Lane 1: Protein ladder
Lane 2-7: Protein bands in the WT
Lane 9-14: Protein bands in the GRKO

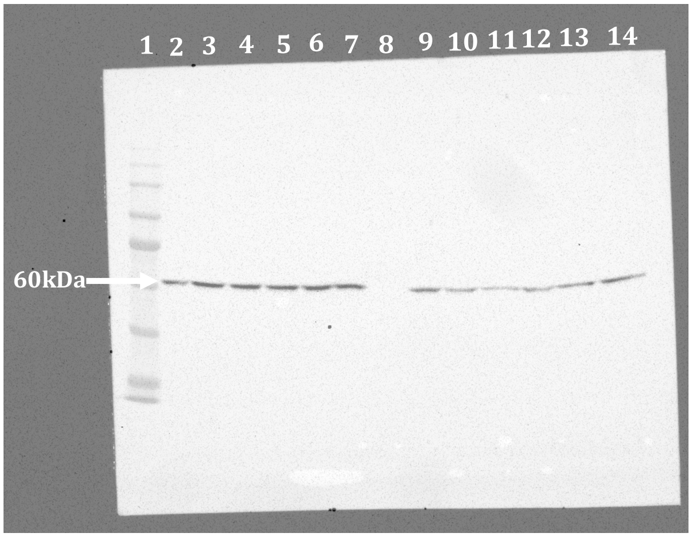

Figure 9C
Total-Akt blot 1
Arrow indicates Total-Akt (60 kDa)
Lane 1: Protein ladder
Lane 2-4: Protein bands in the WT
Lane 6-8: Protein bands in the GRKO
Lane 9: Rainbow trout brain

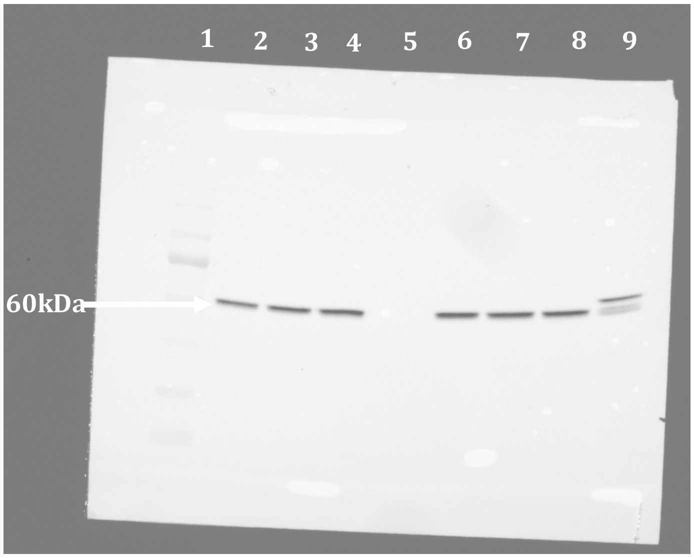

Figure 9C
Total-Akt blot 2
Arrow indicates Total-Akt (60 kDa)
Lane 1: Protein ladder
Lane 2-7: Protein bands in the WT
Lane 9-14: Protein bands in the GRKO

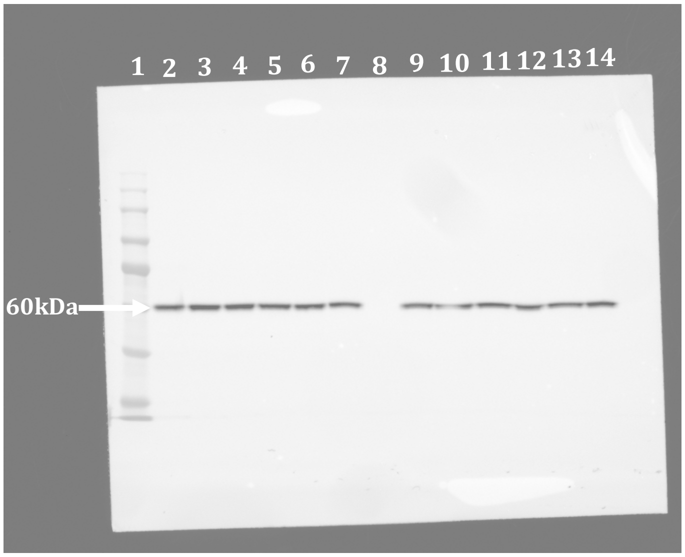

Figure 9E
P-mTOR
Arrow indicates P-mTOR (289 kDa)
Lane 1: Protein ladder
Lane 2-7: Protein bands in the WT
Lane 9-14: Protein bands in the GRKO

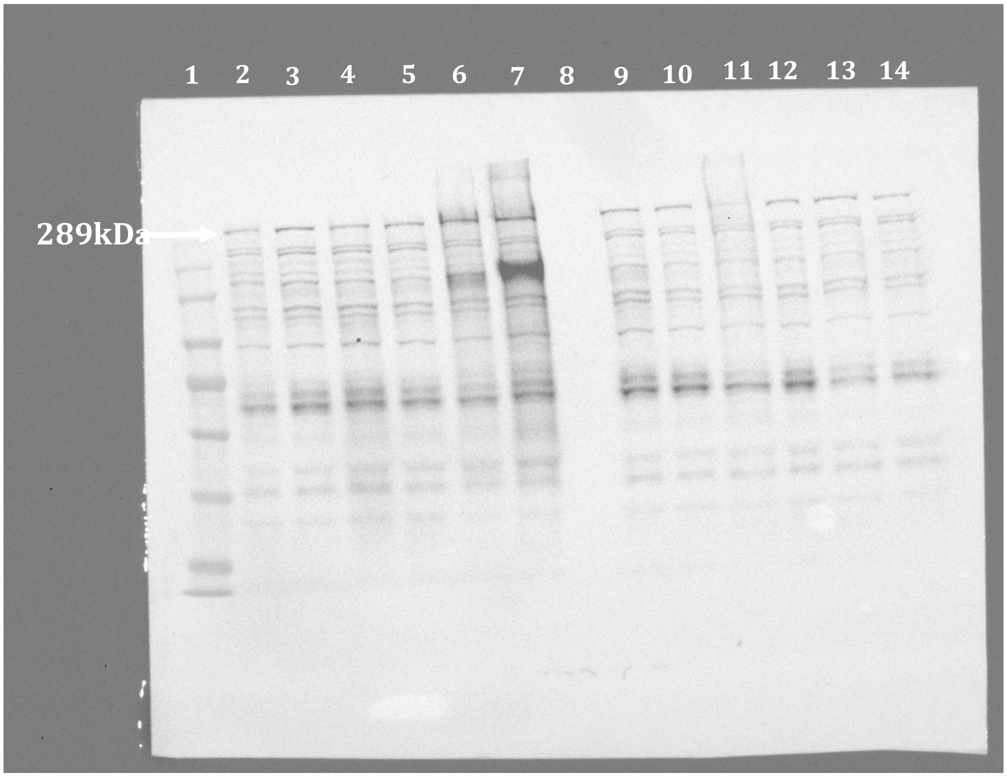

Figure 9E
B-actin
Arrow indicates B-actin (42 kDa)
Lane 1: Protein ladder
Lane 2-7: Protein bands in the WT
Lane 9-14: Protein bands in the GRKO

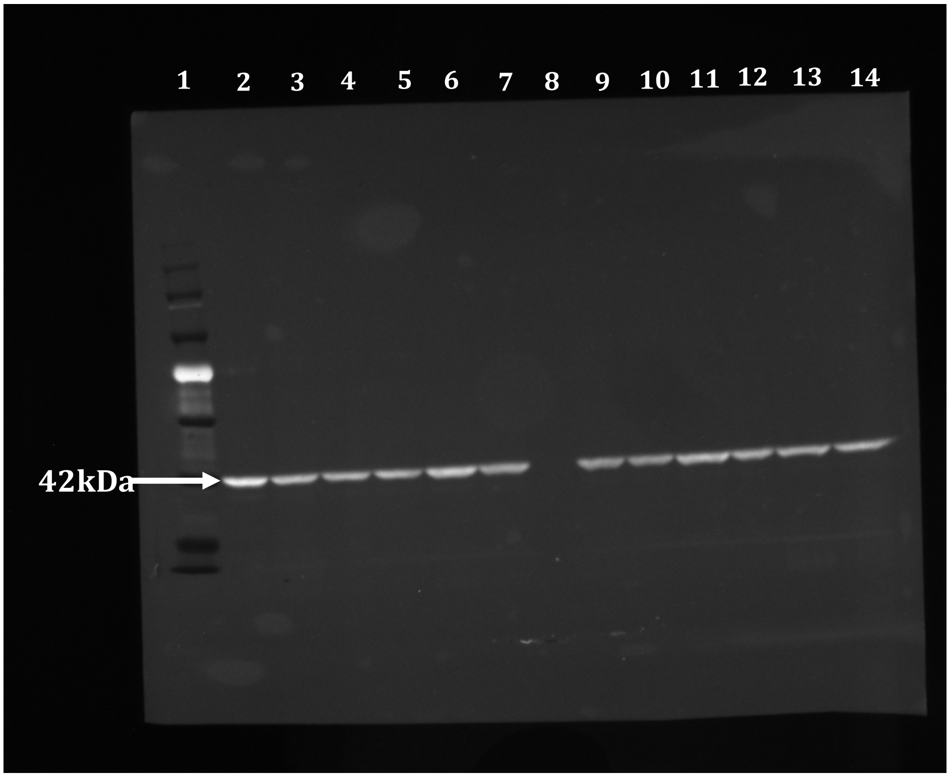

References

1. Faught, E. & Vijayan, M. M. Loss of the glucocorticoid receptor in zebrafish improves muscle glucose availability and increases growth. *Am. J. Physiol.-Endocrinol. Metab.* **316**, E1093–E1104 (2019).
2. Nesan, D. *et al.* Glucocorticoid Receptor Signaling Is Essential for Mesoderm Formation and Muscle Development in Zebrafish. *Endocrinology* **153**, 1288–1300 (2012).
3. Nesan, D. & Vijayan, M. M. Maternal Cortisol Mediates Hypothalamus-Pituitary-Interrenal Axis Development in Zebrafish. *Sci. Rep.* **6**, 22582 (2016).
4. Marvel, M., Spicer, O. S., Wong, T.-T., Zmora, N. & Zohar, Y. Knockout of the Gnrh genes in zebrafish: effects on reproduction and potential compensation by reproductive and feeding-related neuropeptides†. *Biol. Reprod.* **99**, 565–577 (2018).
5. Nesan, D. & Vijayan, M. M. The Transcriptomics of Glucocorticoid Receptor Signaling in Developing Zebrafish. *PLOS ONE* **8**, e80726 (2013).
6. Dalmolin, C., Almeida, D. V., Figueiredo, M. A. & Marins, L. F. Expression profile of glucose transport-related genes under chronic and acute exposure to growth hormone in zebrafish. *Comp. Biochem. Physiol. A. Mol. Integr. Physiol.* **221**, 1–6 (2018).
7. Toyoshima, Y. *et al.* The Role of Insulin Receptor Signaling in Zebrafish Embryogenesis. *Endocrinology* **149**, 5996–6005 (2008).
8. Liu, Q. *et al.* Knockdown of leptin A expression dramatically alters zebrafish development. *Gen. Comp. Endocrinol.* **178**, 562–572 (2012).
9. Feng, Q. *et al.* The Stress-Response Gene redd1 Regulates Dorsoventral Patterning by Antagonizing Wnt/β-catenin Activity in Zebrafish. *PLoS ONE* **7**, e52674 (2012).

10. Rajeswari, J. J., Blanco, A. M. & Unniappan, S. Phoenixin-20 suppresses food intake, modulates glucoregulatory enzymes, and enhances glycolysis in zebrafish. *Am. J. Physiol. - Regul. Integr. Comp. Physiol.* **318**, R917–R928 (2020).
